# Hyaluronan activates Hyal-2/WWOX/Smad4 signaling and causes bubbling cell death when the signaling complex is overexpressed

**DOI:** 10.18632/oncotarget.13268

**Published:** 2016-11-10

**Authors:** Li-Jin Hsu, Qunying Hong, Shur-Tzu Chen, Hsiang-Lin Kuo, Lori Schultz, John Heath, Sing-Ru Lin, Ming-Hui Lee, Dong-Zhang Li, Zih-Ling Li, Hui-Ching Cheng, Gerard Armand, Nan-Shan Chang

**Affiliations:** ^1^ Guthrie Research Institute, Laboratory of Molecular Immunology, Sayre, PA, USA; ^2^ Institute of Molecular Medicine, National Cheng Kung University College of Medicine, Tainan, Taiwan, ROC; ^3^ Department of Medical Laboratory Science and Biotechnology, National Cheng Kung University College of Medicine, Tainan, Taiwan, ROC; ^4^ Department of Cell Biology and Anatomy, National Cheng Kung University College of Medicine, Tainan, Taiwan, ROC; ^5^ Advanced Optoelectronic Technology Center, National Cheng Kung University, Tainan, Taiwan, ROC; ^6^ Graduate Institute of Biomedical Sciences, College of Medicine, China Medical University, Taichung, Taiwan, ROC; ^7^ Glycomed Research Inc., Hastings on Hudson, New York, NY, USA

**Keywords:** hyaluronan, hyaluronidase, Hyal-2, Smad, WWOX

## Abstract

Malignant cancer cells frequently secrete significant amounts of transforming growth factor beta (TGF-β), hyaluronan (HA) and hyaluronidases to facilitate metastasizing to target organs. In a non-canonical signaling, TGF-β binds membrane hyaluronidase Hyal-2 for recruiting tumor suppressors WWOX and Smad4, and the resulting Hyal-2/WWOX/Smad4 complex is accumulated in the nucleus to enhance SMAD-promoter dependent transcriptional activity. Yeast two-hybrid analysis showed that WWOX acts as a bridge to bind both Hyal-2 and Smad4. When WWOX-expressing cells were stimulated with high molecular weight HA, an increased formation of endogenous Hyal-2/WWOX/Smad4 complex occurred rapidly, followed by relocating to the nuclei in 20-40 min. In WWOX-deficient cells, HA failed to induce Smad2/3/4 relocation to the nucleus. To prove the signaling event, we designed a real time tri-molecular FRET analysis and revealed that HA induces the signaling pathway from ectopic Smad4 to WWOX and finally to p53, as well as from Smad4 to Hyal-2 and then to WWOX. An increased binding of the Smad4/Hyal-2/WWOX complex occurs with time in the nucleus that leads to bubbling cell death. In contrast, HA increases the binding of Smad4/WWOX/p53, which causes membrane blebbing but without cell death. In traumatic brain injury-induced neuronal death, the Hyal-2/WWOX complex was accumulated in the apoptotic nuclei of neurons in the rat brains in 24 hr post injury, as determined by immunoelectron microscopy. Together, HA activates the Hyal-2/WWOX/Smad4 signaling and causes bubbling cell death when the signaling complex is overexpressed.

## INTRODUCTION

Hyaluronidases Hyal-1 and Hyal-2 and hyaluronan (HA) are associated with embryonic development, morphogenesis, wound healing, neurodegeneration, and cancer progression [1–4]. Degraded hyaluronan is critical for cancer cell-associated angiogenesis, survival and metastasis [1–4]. Hyal-1 is a lysosomal enzyme that is secreted from cells, and is considered as a tumor suppressor [5]. However, it enhances extravasation and metastasis of many types of cancer cells [6–8]. Hyal-2 is a lysosomal protein and a candidate tumor suppressor [9], and is also anchored on cell surface via glycosylphosphatidylinositol (GPI) [10]. Hyal-2 participates in glycalyx formation [11]. Surface Hyal-2 is a co-receptor with CD44 for HA [1]. The enzymatic activity of Hyal-2 can be induced under acidic environment [12]. Hyaluronidase PH-20 is also a membrane GPI-anchored protein, and is released as a soluble form [1]. PH-20 is expressed in breast and other cancer tissues [1, 13]. We determined that bovine testicular PH-20 increases the susceptibility of murine L929 fibroblasts and prostate LNCaP cancer cells to tumor necrosis factor (TNF or TNF-α) cytotoxicity [14, 15]. PH-20 induces the expression of proapoptotic p53 and WW domain-containing oxidoreductase (WWOX, WOX1, or FOR) [16–19], which contributes in part to the increased TNF sensitivity in murine L929 fibroblasts.

HA signaling via CD44 receptor has been well documented [1–4, 20]. In a non-canonical pathway, transforming growth factor beta (TGF-β) binds membrane Hyal-2 and then recruits tumor suppressors WWOX and Smad4, and the resulting Hyal-2/WWOX/Smad4 induces SMAD-dependent transcriptional activation in the nucleus [18, 21, 22]. Recently, membrane Hyal-2 is shown to bind a 31-amino-acid peptide Zfra (zinc finger-like protein that regulates apoptosis) for leading to the activation of spleen Hyal-2^+^ CD3^−^ CD19^−^ Z cell in memory anticancer response [23]. Hyaluronidases PH-20, Hyal-1 and Hyal-2 induce the expression of tumor suppressor WWOX [16, 17, 24]. Human *WWOX* gene is located on a chromosomal fragile site 16q23 [25, 26]. Loss of heterozygosity (LOH) and alterations of *WWOX* gene have been shown in a variety of cancers [17, 18, 22, 27–31]. WWOX-mediated suppression of cancer cell growth has been established in *Drosophila* [32], in cell lines, and in drug-induced WWOX expression for cancer treatment [33]. Notably, null mutations of *WWOX/Wwox* gene in humans, rats and mice result in severe neural diseases (e.g. microcephaly, seizure, ataxia, etc.), growth retardation, metabolic disorders, and significantly shortening of life span [22, 34–36]. Essential no spontaneous cancer formation has been seen in the newborns [22, 34–36].

WWOX possesses two *N*-terminal WW domains (containing conserved tryptophan residues), a nuclear localization sequence (NLS), and a *C*-terminal short-chain alcohol dehydrogenase/reductase (ADH/SDR) domain [16–18, 22, 27–31]. WWOX undergoes Tyr33 phosphorylation, binds Ser46-phosphorlated p53, and translocates with p53 to the nucleus in response to stress responses, including anisomycin, TNF-α and UV irradiation [37–39], low energy constant light for photoreceptor degeneration [40], and 17β-estradiol [41]. Both p53 and WWOX induce apoptosis synergistically [16, 37–39]. Calcium ionophore and phorbol ester induce WWOX to undergo Ser14 phosphorylation, which leads to terminal maturation of T cell acute lymphoblastic leukemia via IκBα/ERK/WWOX signaling [42].

A portion of cytosolic WWOX is anchored by Hyal-2 or Ezrin onto the cell membrane [21, 43]. Here, we determined that in WWOX-expressing cells, high molecular weight HA induced the formation of WWOX, Hyal-2 and Smads complex, and the complex underwent relocation to the nucleus. The HA-induced signaling of ectopic Smad/Hyal-2/WWOX was confirmed by real time tri-molecular FRET (Förster resonance energy transfer) microscopy [42]. The Smad/Hyal-2/WWOX complex relocated to the nucleus to induce cell death. Meanwhile, a signaling pathway of Smad/WWOX/p53 was also activated but failed to cause cell death. Functional significance of the Hyal-2/WWOX signaling was tested in a traumatic brain injury model in rat, and the signaling revealed a critical role of nuclear Hyal-2 and WWOX in causing apoptosis under stress conditions.

## RESULTS

### HA induction of nuclear accumulation of WWOX, ERK and smads in WWOX-expressing cells

**Figure 1:**
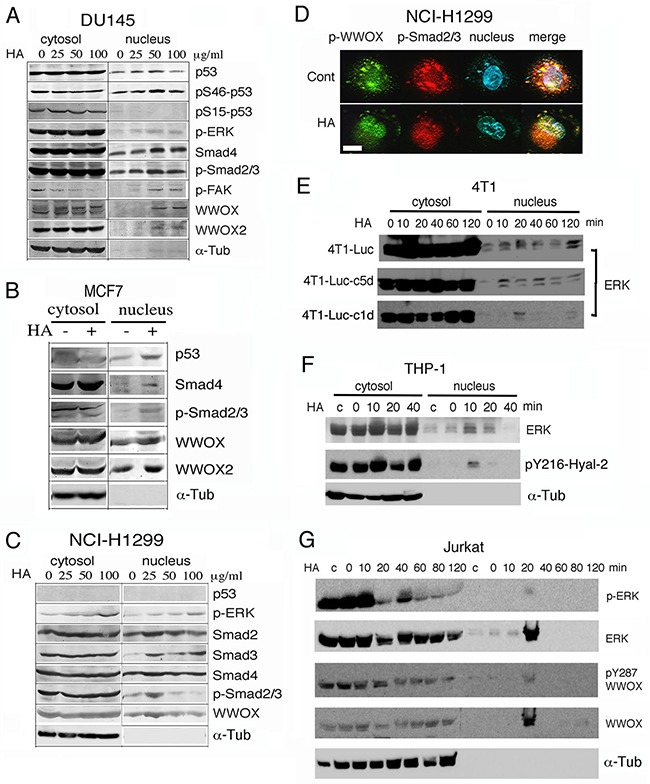
Hyaluronan induces nuclear accumulation of WWOX, Smads and others proteins **A**. Prostate DU145 cells were treated with high-molecular-weight hyaluronan (HA; 10^5^−10^6^ kDa; 50 μg/ml; Sigma) for 1 hr. Nuclear translocation of full length WWOX, isoform WWOX2, p-FAK, p-Smad2/3, Smad4, p-ERK, and pSer46-p53 is shown (~100–300% increases). Time course analysis showed that in response to a low level of HA at 100 ng/ml, WWOX and Smad4 relocated to the nucleus in 40 min (Supplementary Figure S1). **B**. Breast MCF7 cells were treated with HA for 1 hr. Nuclear translocation of WWOX, WWOX2, p53, p-ERK and p-Smad2/3 was shown. **C, D**. p53-deficient NCI-H1299 cells were treated with medical grade HA for 1 hr. Nuclear translocation of WWOX, p-WWOX (Tyr33 phosphorylation) and Smads is shown by Western blotting and fluorescence microscopy (~100–300% increases in nuclear localization using 25 μg/ml HA). 25 μg/ml HA was used in (D). Note the colocalization of p-WWOX with p-Smad2/3 in the cytosol and their nuclear accumulation post HA treatment. Scale bar, 10 μm. **E-G**. Mouse breast cancer 4T1 cells (wild type and 2 cold-resistant variants), human monocytic THP-1 cells, and human leukemia Jurkat T cells were treated with HA (25 μg/ml) for indicated times. Nuclear accumulation of indicated proteins occurred in 10-20 min. c = medium control; α-Tub=α-Tubulin.

HA either enhances or blocks the TGF-β1 signaling [24]. HA binds cell surface CD44 receptor, then activates the closely associated TGF-receptor type I (TβRI), and leads to activation of Smads in triple-negative breast metastatic MDA-MB-231 cells [44]. These cells lack estrogen receptor (ER), progesterone receptor (PR) and Her2/neu [44]. Here, we determined that exposure of malignant prostate DU145 cells to high-molecular-weight hyaluronan (10^5^−10^6^ kDa) for 1 hr resulted in nuclear translocation of full-length WWOX (46 kDa), isoform WWOX2 (42 kDa), p-FAK, p-Smad2/3, Smad4, p-ERK, and pS46-p53 (Figure 1A; ~100-300% increased in nuclear accumulation). In contrast, pS15-p53 did not translocate to the nuclei. Ser46 phosphorylation is essential for p53 to mediate apoptosis [38, 39, 41]. In time-course experiments, HA, as low as 100 ng/ml, caused nuclear accumulation of WWOX, Hyal-2 and Smad4 in 20 min post stimulation (Supplementary Figure S1). Similar results were observed using 25-100 μg/ml HA (data not shown). DU145 cells do not express androgen receptors (AR) [45], but have an intact TGF-β signaling pathway [46] and express mutant p53 [47]. Similarly, HA induced nuclear accumulation of WWOX, WWOX2, p53, p-ERK and p-Smad2/3 in WWOX-expressing breast MCF7 cells during treatment for 1 hr (Figure 1B; ~40-100% increased in nuclear accumulation). MCF7 cells express functional ER [41, 44] and wild type p53 and WWOX [41].

Stimulation of p53-negative lung NCI-H1299 cells with highly purified HA (medical grade from Lifecore) for 1 hr also resulted in nuclear accumulation of WWOX, p-WWOX (Tyr33 phosphorylation), p-ERK and Smads (~100-300% increased), as determined by both Western blotting and immunofluorescence (Figures 1C, 1D). Colocalization of p-WWOX with p-Smad2/3 was shown in the cytoplasm, and HA stimulated translocation of these proteins to the nuclei (Figures 1C, 1D). Yet, a large portion of p-Smad2/3 was present in the cytoplasm (Figures 1C, 1D).

We examined the responsiveness of WWOX-expressing cells to HA in time-course experiments. When murine breast cancer 4T1 cells, including 4T1-Luc, 4T1-Luc-c1d and 4T1-Luc-c5d, were exposed to HA for indicated times, ERK relocated to the nucleus in 10-20 min (Figure 1E). 4T1-Luc-c1d and 4T1-Luc-c5d are cold-resistant variants of 4T1-Luc. Human monocytic THP-1 and U937 cells, were treated with HA (25 μg/ml) for indicated times. Nuclear accumulation of ERK and pY216-Hyal-2 occurred in 10-20 min (Figure 1F; data not shown for U937). We generated antibody against Hyal-2 phosphorylation at Tyr216. The quality of this antibody is also shown (Figure 4A). Human leukemia Jurkat and Molt-4 T cells were also treated with HA, which led to nuclear accumulation of ERK, p-ERK and pY287-WWOX in the nuclei in 20 min (Figure 1G; data not shown for Molt-4). Production of antibody against pY287-WWOX has been described [42]. Similar results were observed using TβRII-deficient WWOX-expressing HCT116 cells [21] (data not shown).

### WWOX-negative cells are refractory to HA-induced nuclear relocation of Smad4 and other proteins

**Figure 2:**
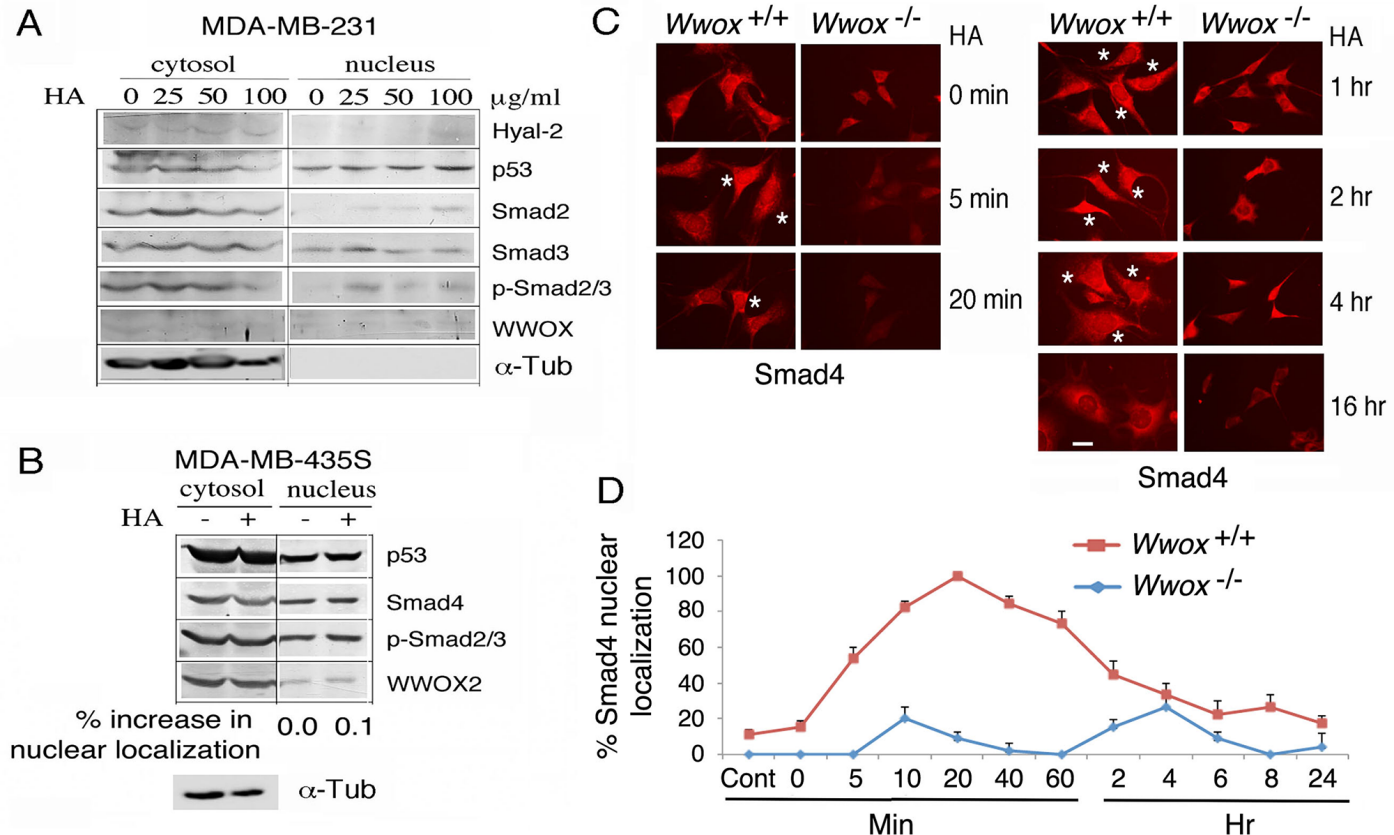
Wild type WWOX is necessary for HA induction of protein nuclear translocation **A, B**. WWOX-deficient breast MDA-MB-231 and MDA-MB-435S were treated with high-molecular-weight HA (25 μg/ml) for 1 hr. Nuclear translocation of each indicated protein was retarded (<10% increase in nuclear localization compared to controls). **C, D**. Exposure of wild type *Wwox*^+/+^ MEF cells to HA (25 μg/ml) resulted in relocation of endogenous Smad4 into the nucleus in 5 min (also see Supplementary Information Figures S3 and S5). When knockout *Wwox*^−/−^ MEF cells were stimulated with HA, Smad4 appeared to relocate into nucleus in 4 hr (also see Supplementary Figures S2-S4). The extent of Smad4 nuclear localization was quantified (n=4; 20 cells per count). Scale bar, 10 μm.

By using WWOX-negative cells, we determined that these cells are refractory to HA-induced accumulation of Smads, p53, and other proteins of interest in the nucleus. Unlike MCF7, triple negative MDA-MB-231 and MDA-MB-435S cells express little or no wild type WWOX, but MDA-MB-435S has WWOX2 expression [41]. Both MDA-MB-231 and MCF7 cells are responsive to TGF-β1-mediated growth suppression [48]. Treatment of MDA-MB-231 with HA did not effectively induce accumulation of p53 and Smad2/3 in the nucleus (Figure 2A; less than 10% for each indicated protein compared to the levels at time zero). Similarly, MDA-MB-435S cells were not responsive to HA-mediated nuclear translocation of WWOX2, p53, Smad4, and p-Smad2/3 (Figure 2B).

By immunofluorescence microscopy, HA induced relocation of a portion of endogenous Smad4 into the nuclei of wild type *Wwox*^+/+^ mouse embryonic fibroblast (MEF) cells in 5 min (see stars in Figure 2C), and the nuclear Smad4 appeared to migrate out to the cytoplasm 6 hr later (Figures 2C, 2D and Supplementary Information Figures S2 and S4). In stark contrast, when knockout *Wwox*^−/−^ MEF cells were stimulated with HA, endogenous Smad4 did not appear to relocate into nucleus (Figures 2C, 2D and Supplementary Information Figures S3 and S4). Presence of Smads in the nucleus of the aforementioned test cells is probably due to activation of the canonical TGF-β pathway. A recent study showed that WWOX binds and sequesters Smad3 in the cytoplasm and thereby controls its transcriptional activity in the nucleus [49]. Loss of WWOX allows nuclear accumulation of Smads and other transcription factors to promote cancer growth [22, 27, 28, 49]. Overall, HA-induced protein nuclear accumulation is a universal phenomenon in cells, and is not related with the status of TβRII, ER, AR and p53.

### Blebbistatin inhibits HA-induced relocation of WWOX, ERK and JNK to the nucleus

**Figure 3:**
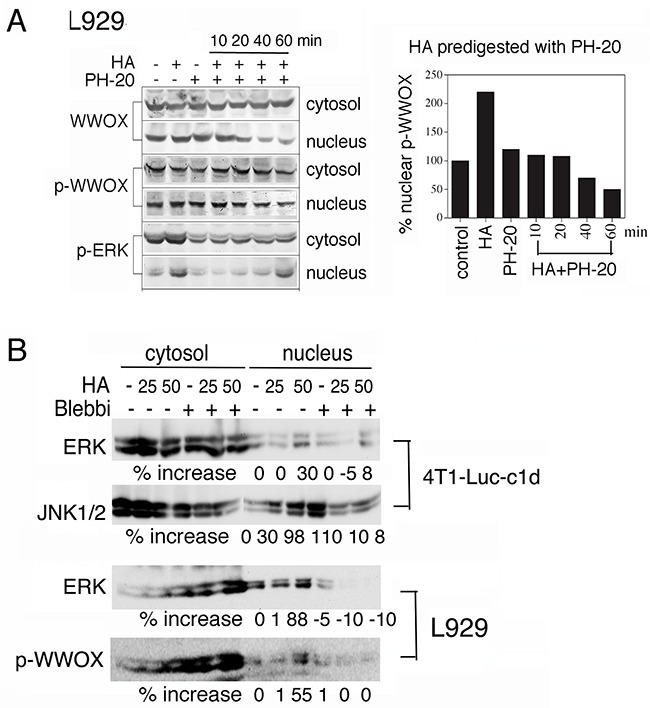
Blebbistatin blocks HA-mediated protein nuclear translocation **A**. HA (100 μg/ml) was predigested with PH-20 (100 units/ml) for various indicated times. The digested HA could not induce nuclear translocation of WWOX, p-WWOX and p-ERK in L929 cells (representative data from 2 experiments). The extent of p-WWOX nuclear translocation is quantified (right panel). **B**. L929 and 4T1-Luc-c1d cells were pre-treated with blebbistatin (10 μM) for 1 hr, followed by exposure to HA (25 and 50 μg/ml) for determining the relocation of indicated proteins to the nucleus by Western blotting. Blebbistatin blocked HA-induced nuclear relocation of ERK, JNK and pY33-WWOX. p-WWOX = pY33-WWOX; Blebbi = blebbistatin.

When native HA was pre-digested with hyaluronidase PH-20, the degraded HA did not induce nuclear translocation of WWOX, p-WWOX, and p-ERK in L929 cells (Figure 3A). Similar results were observed in MCF7 and other WWOX-expressing cells (data not shown). Digestion of HA with *Streptomyces hyalurolyticus* hyaluronidase produced similar results. These observations suggest that integrity of HA is critical for the Hyal-2/WWOX/Smads signaling in cells. Alternatively, high molecular weight HA appears to exert mechanical stress to cell membrane, so that Hyal-2 may undergoes internalization to signal with WWOX and Smads. Cells were pre-exposed to blebbistatin, an inhibitor of myosin II, to relieve membrane stress, followed by treating with HA for 1 hr. Blebbistatin blocked HA-induced nuclear accumulation of ERK, JNK and pY33-WWOX in 4T1-Luc and L929 cells (Figure 3B).

HA did not effectively cause relocation of cytosolic Smad3 to the nuclei (Figures 1A, 1D). In contrast to the aforementioned observations (Figure 3B), blebbistatin rapidly induced accumulation of Smad3 in the nucleus of DU145 cells and HA reversed the effect (Supplementary Figure S5). However, both HA and/or blebbistatin failed to induce accumulation of Smad3 in the nuclei in the wild type *Wwox*^+/+^ MEF cells (data not shown).

### HA induces nuclear accumulation of Hyal-2

**Figure 4:**
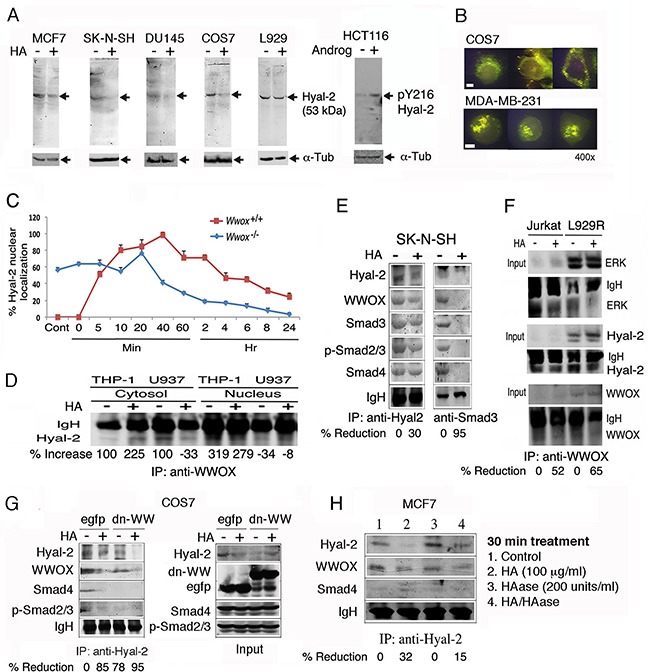
HA increases the complex formation of Hyal-2, WWOX and Smads, followed by reduction **A**. Indicated cell lines were treated with HA (50 μg/ml) for 30 min at 37°C. Cytosolic fractions were used for reducing SDS-PAGE and Western blotting. HCT116 cells were treated with androgen (100 ng/ml) for 30 min, which led to increased pY216-Hyal-2 expression. **B**. Immunostaining of non-permeabilized COS7 cells shows the expression of Hyal-2 on cell surface. Hyal-2 is clustered on the surface of non-permeabilized MDA-MB-231 cells. Scale bar, 5 μm. **C**. HA (25 μg/ml) rapidly induced translocation of Hyal-2 to the nucleus in the wild type *Wwox^+/+^* MEF cells, followed by reduction. In the knockout *Wwox^−/−^* MEF cells, approximately 60% of endogenous Hyal-2 is present in the nucleus. HA reduces the nuclear localization. **D**. By immunoprecipitation using WWOX antibody, HA increased the WWOX/Hyal-2 complex in the cytosol, whereas the nuclear level of the complex was still low in THP-1 cells. In U937 cells, HA reduced the cytosolic WWOX/Hyal-2 complex in 30 min and showed the increased complex in the nucleus. **E**. HA (25 μg/ml) reduced the cytosolic Hyal-2/WWOX/Smads complex in SK-N-SH cells in 30 min, as determined by immunoprecipitation using Hyal-2 antibody. Immunoprecipitation by Smad3 antibody revealed the presence of the Hyal-2/WWOX/Smads complex in resting cells and HA decreased the complex. **F**. Jurkat T cells and L929R fibroblasts were treated with HA (25 μg/ml) for 30 min, followed by processing immunoprecipitation with WWOX antibody. HA reduced the complex formation of cytosolic WWOX/Hyal-2/ERK. **G**. HA reduced the complex formation of Hyal-2/WWOX/Smad4 in EGFP-expressing COS7 cells. Also, ectopic EGFP-dn-WW suppressed the complex formation. In the input, one-tenth amounts of the cell lysates were loaded in the SDS-PAGE. **H**. The Hyal-2/WWOX/Smad4 complex was not affected by hyaluronidase PH-20 treatment of MCF7 cells for 1 hr.

We determined whether WWOX participates in HA-induced nuclear translocation of Hyal-2. By using generated polyclonal antibodies against Hyal-2 [21, 24], a 53-kDa protein is shown in breast cancer MCF7, neuroblastoma SK-N-SH, prostate cancer DU145, kidney fibroblast COS7 and L929 fibrosarcoma cells (Figure 4A). Exposure of these cells to HA reduced the expression of cytosolic Hyal-2 by approximately 50-90% (Figure 4A). We also produced antibody against Hyal-2 phosphorylation at Tyr216. Androgen upregulated the expression of pY216-Hyal-2 in colon cancer HCT116 cells (Figure 4A). To further confirm the membrane localization of Hyal-2 [28, 31], immunostaining of non-permeabilized COS7 cells showed the presence of cell surface Hyal-2 (Figure 4B). Nuclear staining with DAPI failed to reveal the nuclei (data not shown), indicating the integrity of these cells. Hyal-2 is clustered, in part, on the cell surface of COS7 and MDA-MB-231 cells (Figure 4B). In permeabilized HCT116, H1299, MCF7, DU145, COS7, L929 and other cells, Hyal-2 was also present on the cell membrane (data not shown) and in the cytoplasm [9, 21, 24]. We have shown that Hyal-2 translocates from the lysosomes to the mitochondria during staurosporine-mediated apoptosis, suggesting that Hyal-2 participates in damaging to mitochondria during apoptosis [24].

By stimulating wild type *Wwox*^+/+^ MEF cells with HA, endogenous Hyal-2 rapidly translocated to the nucleus in 5 min (Figure 4C). Interestingly, endogenous Hyal-2 mainly localized in the nucleus of knockout *Wwox*^−/−^ MEF cells (~60%). Reduction of nuclear accumulation of Hyal-2 occurred in 20-40 min post HA stimulation (Figure 4C). The observations suggest that WWOX limits relocation of Hyal-2 to the nucleus.

### HA increases the complex formation of Hyal-2, WWOX and Smads, followed by reduction

We examined the complex formation between Hyal-2 and WWOX. By immunoprecipitation using WWOX antibody, HA increased the binding of WWOX with Hyal-2 in the cytosol in 30 min in THP-1 cells, whereas at this point the complex remained in the cytosol and no apparent translocation to the nucleus was shown (Figure 4D). In contrast, HA induced nuclear translocation of the WWOX/Hyal-2 complex in U937 cells, and the level of the complex in the cytoplasm was reduced (Figure 4D). In resting SK-N-SH cells, there was a cytosolic Hyal-2/WWOX/Smads complex, whereas HA reduced the level of the complex, as determined by co-immunoprecipitation using Hyal-2 antibody (Figure 4E). The reduction is due to relocation of Hyal-2 to the nucleus. In parallel, immunoprecipitation by Smad3 antibody revealed the presence of the Hyal-2/WWOX/Smads complex. However, due to nuclear relocation, co-immunoprecipitation by Smad3 antibody failed to show the Hyal-2/WWOX/Smads complex (Figure 4E).

Similarly, WWOX bound Hyal-2 and ERK in resting Jurkat T and TNF-resistant L929R cells, and HA reduced the WWOX/Hyal-2/ERK complex formation in the cytosol in 30 min, suggesting nuclear translocation of the complex (Figure 4F). Non-immune sera did not precipitate the aforementioned complexes (data not shown). We have recently demonstrated the binding interaction between WWOX with ERK and IκBα [42]. Similar results were observed with THP-1 and U937 monocytic cells and TNF-sensitive L929 cells (data not shown).

A plasmid construct expressing an EGFP-tagged dominant negative WWOX with alterations at the *N*-terminal WW domain (dn-WW) was made [37, 39]. The dominant negative suppresses stress stimuli-induced Tyr33-phosphorylation in WWOX and Ser46-phosphoryltaion in p53, and p53/WWOX-mediated apoptosis [37, 39]. By immunoprecipitation using anti-Hyal-2 antibody, we demonstrated the binding of Hyal-2 with WWOX, Smad4 and p-Smad2/3 in the control EGFP-expressing COS7 cells (Figure 4G). Ectopic dn-WW reduced the binding of Smads and WWOX with Hyal-2 (Figure 4G), suggesting that dn-WW reduces Tyr33 phosphorylation in WWOX to cause dissociation. Again, HA dissociated the Hyal-2/WWOX/Smad4 complex (Figure 4G). In MCF7 cells, hyaluronidase PH-20 alone did not abolish the Hyal-2/WWOX/Smads complex, and that hyaluronidase-degraded HA had a reduced effect on the complex (Figure 4H).

### Yeast two-hybrid analysis for determining domain/domain binding for the WWOX/Hyal-2/Smad4 complex

**Figure 5:**
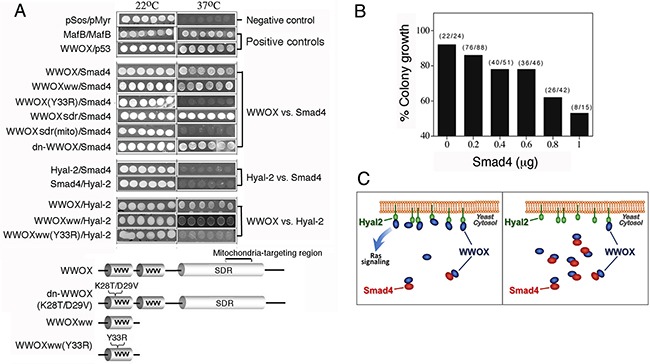
Tyr33 phosphorylation of WWOX is essential for interacting with Smad4 and Hyal-2, and Smad4 competitively blocks the binding of WWOX to membrane Hyal-2 **A**. Ras rescue-based protein/protein interaction in the cytoplasm by yeast two-hybrid was performed [16, 21, 37, 39]. In positive controls, binding of WWOX with p53 and MafB self-interaction are shown, as evidenced by the growth of yeast at 37°C using a selective agarose plate containing galactose. No yeast growth at 37°C was observed for the empty pSos/pMyr vectors in negative controls. The *N*-terminal first WW domain of WWOX (WWOXww) bound Smad4 and Hyal-2. Alteration of Tyr33 to Arg33 in the first WW domain, WWOXww(Y33R), abolished its interaction with Smad4 and Hyal-2. Dominant negative WWOX, dn-WWOX(K28T/D29V), also bound Smad4. The mitochondria-targeting area (amino acid 209-273) of the *C*-terminal SDR domain did not bind Smad4. No direct interaction was observed for Hyal-2 and Smad4. **B, C**. In this competitive binding assay, yeast cells were transfected with Hyal-2 (target; green) for anchoring onto cell membrane, in the presence of a constant amount of WWOX (bait; dark blue) and various amounts of Smad4 (competitor; red). % Colony growth = (survival colonies at 37°C) / (total colonies grown at 22°C) (see brackets). Smad4 competitively blocked the binding of WWOX to membrane Hyal-2, and thereby prevented yeast growth.

To further substantiate the above observations, we mapped the domains in the WWOX that interacts with Smad4 and Hyal-2. By utilizing Ras rescue-based yeast two-hybrid analysis [16, 21, 37, 39, 42], Smad4 was anchored onto the cell membrane as target, and various WWOX constructs were expressed in the cytoplasm as baits. WWOX physically bound Smad4 via its *N*-terminal first WW domain (Figure 5A), as evidenced by the growth of yeast at 37°C using a selective agarose plate containing galactose. No binding interaction was observed using a phosphorylation mutant of WW domain, WWOXww(Y33R), suggesting an essential role of Y33 phosphorylation in the binding. The *C*-terminal SDR domain of WWOX also bound Smad4, whereas the mitochondria-targeting region in SDR domain did not bind Smad4 (Figure 5A). dn-WWOX, with alterations at K28 and D29 (K28T/D29V) [37], was capable of binding Smad4 (Figure 4), suggesting that Smad4 binds both the SDR domain and the pY33-WW domain. Compared to wild type murine Smad4 (60 kDa), our truncated Smad4 (43 kDa) has a *C*-terminal deletion from amino acid #392 to 551 (Genbank accession AY493561) [21].

In agreement with our previous report [21], the first WW domain of WWOX bound Hyal-2 (Figure 5A). Alteration of the conserved Tyr33 phosphorylation site abolished the binding (Figure 5A). In contrast, Hyal-2 could not bind Smad4 (Figure 5A), indicating that WWOX connects the binding of Hyal-2 with Smad4. Results from co-immunoprecipitation support these observations (Figures 4D-4G). As a negative control, no binding interactions were observed for the empty pSos/pMyr vectors. In positive controls, WWOX/p53 binding and MafB self-interaction were observed (Figure 5A).

### Hyal-2 competes with Smad4 in binding to WWOX

The aforementioned results revealed that both Hyal-2 and Smad4 bound to the *N*-terminal pY33-WW domain in WWOX, although Samd4 can also bind the C-terminal SDR domain (Figure 5A). To test whether there is a competitive binding between Hyal-2 and Smad4 to WWOX, yeast cells were transfected with membrane-anchored Hyal-2 (target), in the presence of a fixed amount of WWOX (bait) and various amounts of Smad4 (competitor) in the cytoplasm. Smad4 competitively blocked the binding of Hyal-2 with WWOX (Figures 5B, 5C). That is, once the p-WWOX/Smad4 complex is formed in the cytoplasm, the complex may have a reduced binding for membrane Hyal-2 and thereby prevents yeast cell growth at 37°C (Figures 5B, 5C).

### dn-WWOX blocks HA-induced nuclear accumulation of Smads

To further verify the observations that WWOX connects the binding interaction between Hyal-2 and Smads, dn-WWOX was utilized to block the WWOX function. COS7 cells were transfected with the expression construct of EGFP-dn-WWOX or EGFP by electroporation, cultured 48 hr, and then treated with HA for 30 min. dn-WWOX blocked the HA-induced nuclear translocation of p-Smad2/3 and Smad4 (Supplementary Figure S6).

### Agonist anti-Hyal-2 antibody and antisense Hyal-2 mRNA stimulate nuclear translocation of WWOX and Smads

**Figure 6:**
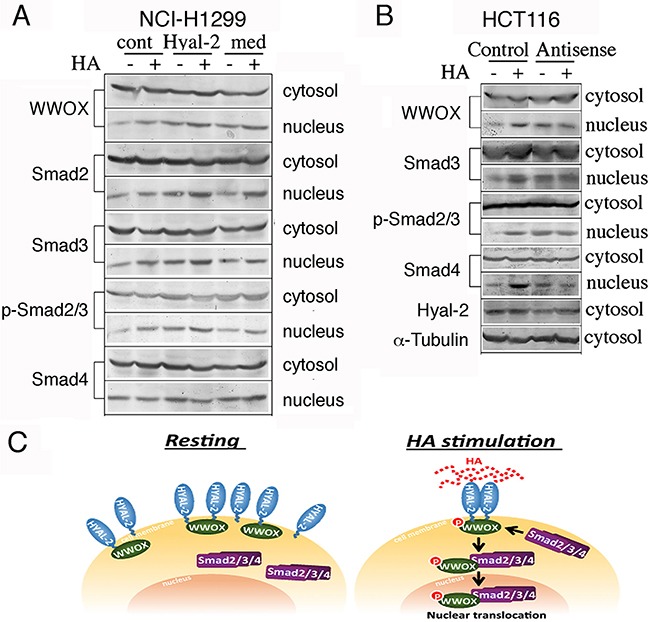
Induction of WWOX and Smads nuclear accumulation by agonist Hyal-2 antibodies and antisense mRNA **A**. Compared to non-immune serum (cont) and medium control (med), exposure of NCI-H1299 cells to agonist antibodies against Hyal-2 (1:1000 dilution) for 30 min resulted in spontaneous nuclear translocation of WWOX and Smads (~75-200% increase in nuclear localization; see the nuclear protein levels *without* HA treatment; representative data from 2 experiments). These cells were further exposed to HA (100 μg/ml) for 30 min. **B**. Similarly, suppression of Hyal-2 expression by antisense mRNA (~70% reduction) spontaneously induced nuclear translocation of WWOX and Smads in TβRII-deficient colon HCT116 cells. Without Hyal-2, HA could not increase nuclear translocation of these proteins (~100-200% increase in nuclear localization; see the nuclear protein levels *without* HA treatment; representative data from 2 experiments). **C**. In resting cells, Hyal-2 binds WWOX in the cell membrane or in the lysosome. HA increases the formation of the Hyal-2/WWOX/Smads complex for relocating to the nucleus. Without WWOX, Hyal-2 may spontaneously accumulate in the nucleus.

Whether Hyal-2 antibody could induce activation of WWOX and Smads was examined. Exposure of NCI-H1299 cells to diluted anti-Hyal-2 antiserum for 30 min resulted in spontaneous nuclear translocation of WWOX and Smads (Figure 6A). In contrast, non-immune serum and culture medium had no effects (Figure 6A). These results were further confirmed with L929 and HCT116 cells (data not shown). Furthermore, suppression of Hyal-2 expression by antisense mRNA spontaneously induced nuclear translocation of WWOX and Smads in TβRII-deficient HCT116 cells (Figure 6B). Without Hyal-2, HA could not further induce nuclear translocation of WWOX and Smads (Figure 6B). These results were also observed when other types of cells were tested (data not shown). Taken together, HA increases the complex formation of Hyal-2 with pY33-WWOX and Smads (Figure 6C). The Hyal-2/WWOX/Smads complex relocates to the nucleus. While a portion of WWOX and Hyal-2 are in the lysosome, formation of the Hyal-2/WWOX/Smads complex is expected to occur in response to HA.

### Time-lapse FRET microscopy of HA-induced signaling involving Smad4/WWOX/p53 for membrane blebbing and Smad4/Hyal-2/WWOX for bubbling cell death

**Figure 7:**
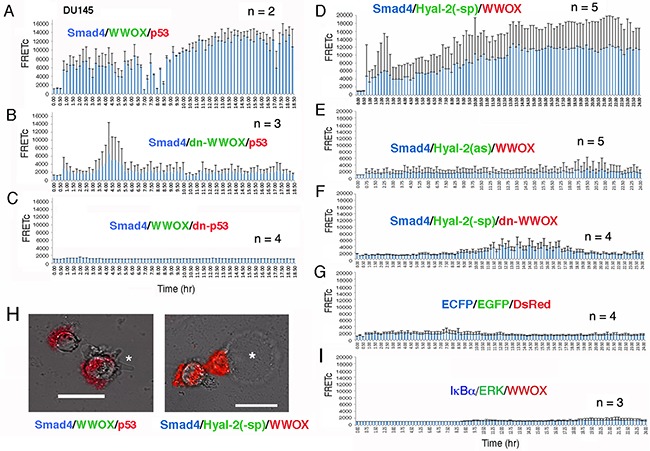
Time-lapse FRET microscopy for HA-activated signaling pathways **A**. DU145 cells were transiently transfected with ECFP-Smd4, EGFP-WWOX and DsRed-monomer-p53 expression constructs. HA (25 μg/m) induced energy transfer from ECFP-Smad4 to EGFP-WWOX and then to DsRed-monomer-p53 for the Smad4/WWOX/p53 signaling. Data are shown as FRETc (FRET concentration). **B, C**. Dominant negatives for WWOX and p53 abolished the signaling. **D**. HA also induced signaling for ECFP-Smd4, EGFP-Hyal-2(−sp) and DsRed-monomer-WWOX. Cytosolic EGFP-Hyal-2(−sp) is devoid of the GPI linkage. **E, F**. Both antisense mRNA targeting Hyal-2 and dn-WWOX abolished the HA-induced signaling. **G**. In negative controls, HA did not induce signaling in cells expressing ECFP, EGFP and DsRed. **H**. The Smad4/WWOX/p53-expressing cells started to undergo membrane blebbing post stimulation with HA for 1.5 hr (see the star in the left). No cell death occurred. In contrast, Smad4/Hyal-2/WWOX-expressing cells underwent bubbling cell death [51, 52], post HA stimulation for 5-16 hr (see the star for the generated bubble in the right). Also, see Supplementary Video legends and Videos S1–S9. **I**. Similarly, HA failed to induce the activation of the IκBα/ERK/WWOX signaling [42].

We examined HA-induced signaling by time-lapse tri-molecular FRET microscopy [42]. DU145 cells were transfected with ECFP-Smad4, EGFP-WWOX and DsRed-monomer-p53 expression constructs. The energy emitted from ECFP goes to EGFP and finally to DsRed monomer. That is, ECFP-Smad4 was excited to allow the energy transfer down to EGFP-WWOX and then to DsRed-monomer-p53. WWOX acts as a bridge for Smad4 and p53 in the energy transfer or signaling. Positive signals were observed in HA-stimulated DU145 cells for the Smad4/WWOX/p53 signaling (Figure 7A and Supplementary Video 1). By merging the resulting FRET signals with the bright field imaging, we found that cells exhibited membrane blebbing without cell death (see star in the left panel, Figure 7H and Supplementary Video 2). Both dn-WWOX and dn-p53 abolished the signaling event (Figures 7B, 7C and Supplementary Videos 3 and 4). We have previously reported that membrane blebbing is an occurrence for cells to resist death, rather than committing them to die [50].

In another signaling, HA activated the signaling of ECFP-Smad4, EGFP-Hyal-2(−sp) and DsRed-monomer-WWOX (Figure 7D and Supplementary Video 5). EGFP-Hyal-2(−sp) is devoid of the GPI linkage and is for intracellular expression. Notably, HA-treated cells underwent bubbling cell death (see star in the right panel, Figure 7H and Supplementary Video 6). We have recently reported UV/cold shock-induced non-apoptotic bubbling cell death – one bubble per cell independently of caspase activation [51, 52]. Antisense mRNA-targeting Hyal-2 abolished the signaling (Figure 7E and Supplementary Video 7). Also, dn-WWOX abolished the singling event (Figure 7F and Supplementary Video 8). In negative controls, HA failed to induce signaling in cells expressing ECFP, EGFP and DsRed (Figure 7G and Supplementary Video 9). We have recently demonstrated that calcium ionophore A23187 and phorbol myristate ester activates the signaling of IκBα/ERK/WWOX in lymphocytic cells [42]. However, HA was not able to activate the IκBα/ERK/WWOX signaling (Figure 7H and Supplementary Video 10).

### Immunoelectron microscopy of HA-induced nuclear relocation of WWOX and Smad4

**Figure 8:**
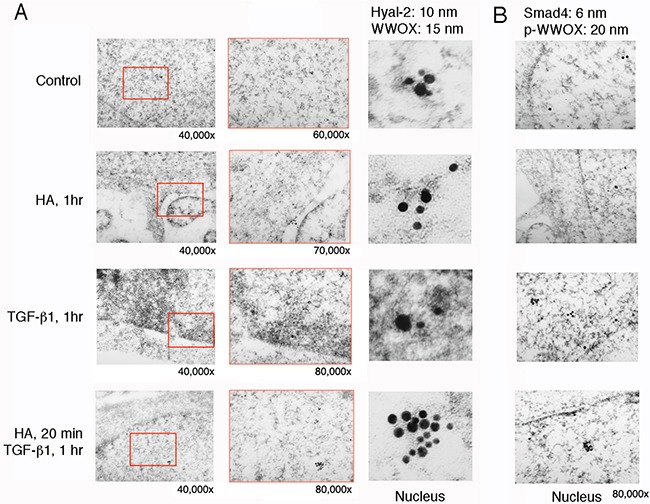
Immunoelectron microscopy analysis for HA induction of WWOX/Hyal-2 nuclear translocation **A**. HCT116 cells were treated with HA for 20 min and then subjected to processing for immunoelectron microscopy using antibodies against WWOX and Hyal-2, plus indicated immunogold particles. Where indicated, the enlarged areas were from 40,000 to 80,000x magnifications. Shown in the column at right are digitally enlarged immunogold particles in the nuclei. **B**. Immunostaining for p-WWOX and Smad4 particles is shown.

When HCT116 cells were stimulated with HA for 60 min, both WWOX and Samd4 were shown to localize in the nucleus, as determined by immunoelectron microscopy [21] (Figure 8). However, little or no Hyal-2 was found (Figure 8). Indeed, without using Hyal-2 antibody, HA had a much less activity in inducing nuclear accumulation of WWOX, Hyal-2 and Smad4 in HCT116 cells, as determined by Western blotting (data not shown). TGF-β1 induced nuclear localization of WWOX and Smad4 in HCT116 cells, and the nuclear level of Hyal-2 was low also (Figure 8). Pre-stimulation of cells with HA for 20 min, followed by stimulating with TGF-β1 for 1 hr, resulted in a dramatic increase in the complex formation of WWOX, Hyal-2 and Smad4 in the nucleus (Figure 8). In agreement with our previous observations [21], TGF-β1 participates in the activation of the WWOX/Hyal-2/Smad4 complex.

### Hyal-2, WWOX and Smad4 induce apoptosis synergistically, and suppression of WWOX by siRNA abrogates Smad4-mediated apoptosis

**Figure 9:**
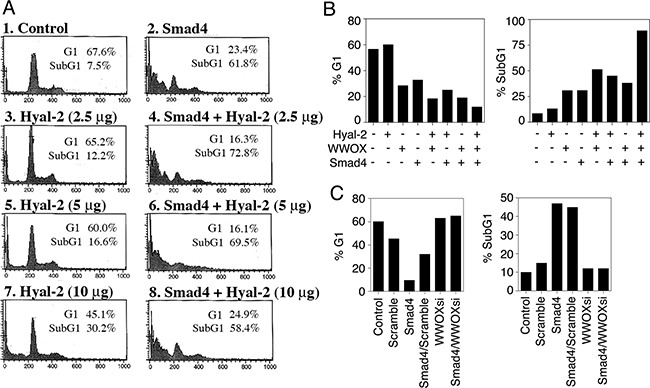
Hyal-2, WWOX and Smad4 synergistically induce apoptosis **A**. L929 cells were electroporated with Hyal-2 and/or Smad4 constructs, cultured 48 hr, and subjected to cell cycle analysis by FACS. Hyal-2 could not bind Smad4 (see Figure 5), and did not enhance the apoptotic function of Smad4 (% apoptosis = % subG1 phase of the cell cycle). **B**. Under similar conditions, L929 cells were transfected with Hyal-2, WWOX, and/or Smad4. Hyal-2 enhanced WWOX-mediated apoptosis. In combination, these 3 proteins increased cell death. **C**. In contrast, WWOXsi blocked Smad4-mediated apoptosis. In controls, scramble siRNA had no effect. WWOXsi, siRNA-targeting WWOX. All data are average from two experiments B, C. Unless otherwise indicated, 5 μg DNA constructs were used in electroporation. Data for both G1 and SubG1 phases are shown for all experiments.

Both WWOX and Smad4 are tumor suppressors and proapoptotic proteins [17, 21, 27–31, 37]. We determined whether ectopic Hyal-2, Smad4, and WWOX induce cell death synergistically. L929 cells were electroporated with Hyal-2, WWOX, and/or Smad4 DNA constructs and cultured 48 hr, followed by cell cycle analysis using flow cytometry. As determined above, Hyal-2 could not directly bind Smad4 (Figure 5). As expected, Hyal-2 could not effectively enhance the apoptotic function of Smad4 (Figure 9A). The extent of apoptosis and growth suppression was measured at the subG1 and G1 phases of the cell cycle, respectively. In agreement with our previous observations [15], Hyal-2 enhanced WWOX-mediated apoptosis, up from 30% to 60% (Figure 9B). When in combination, Hyal-2, WWOX and Smad4 greatly increased the cell death (Figure 9B). The inverse relationship in the G1 phase, as opposed to SubG1 phase, was also observed (Figure 9B).

We have made plasmid constructs expressing small-interfering RNA (siRNA)-targeting WWOX (WWOXsi in pSuppressorNeo) [41]. Ectopic WWOXsi blocked Smad4-mediated growth inhibition and apoptosis (Figure 9C). In controls, scramble siRNA had no effect (Figure 9C). Opposite effect was also seen in the G1 phase. These observations suggest that WWOX acts synergistically with Smad4 in causing apoptosis. In parallel, ectopic WWOX and Hyal-2 enhance the transcription activation of Smad4 for SMAD-regulated promoter, thereby inducing apoptosis [21].

### Traumatic brain injury involves in nuclear accumulation of Hyal-2 and WWOX

**Figure 10:**
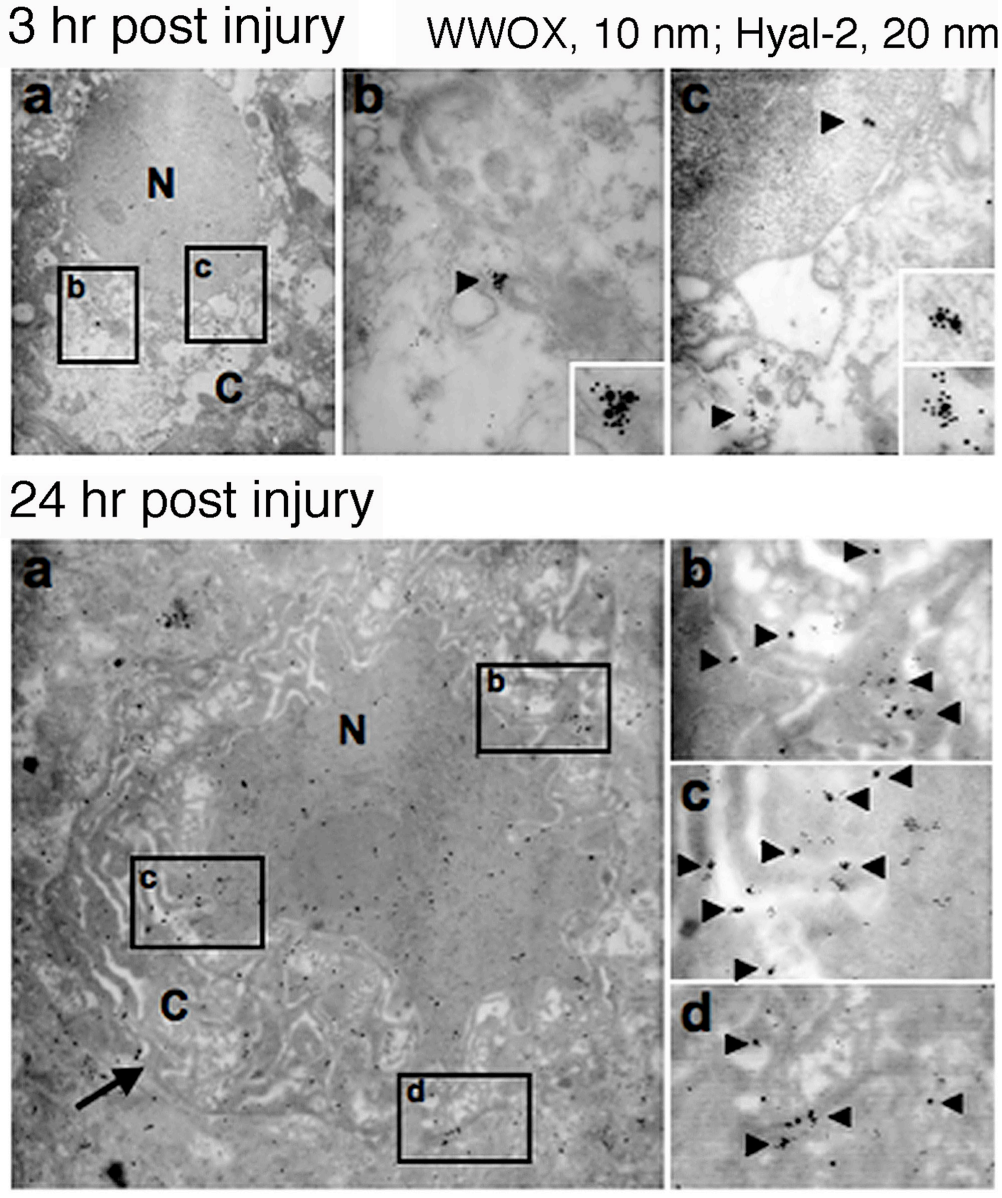
Nuclear accumulation of Hyal-2 and WWOX in apoptotic nuclei of cortical neurons during traumatic brain injury Rats were stabbed with needles into their brains to induce traumatic brain injury. Post injury for 3 and 24 hr, rats were sacrificed. There were increased numbers of apoptotic neurons in the brain cortex, with the presence of the Hyal-2/WWOX complex in the apoptotic nuclei of neurons.

Finally, we determined the potential role of WWOX and Hyal-2 in apoptosis *in vivo*. In a traumatic brain injury, rats were pierced with needles into their brains [40, 53]. We determined that post injury for 3 and 24 hr, there were increased numbers of apoptotic neurons in the brain cortex. And, the complex formation of WWOX/Hyal-2 was increased with time and accumulated in the apoptotic nuclei of neurons (Figure 10).

## DISCUSSION

We have previously determined that TGF-β1 induces the signaling of the Hyal-2/WWOX/Smad4 complex for transcriptional activation of SMAD-responsive element [21]. When the SMAD-responsive element is overly activated, cell death occurs [21]. In this study, we determined by yeast two-hybrid analysis that WWOX acts as a bridge to bind and connect both Hyal-2 and Smad4. Hyal-2 binds to the *N*-terminal Tyr33-phosphorylated first WW domain, and Smad4 binds to the SDR domain. Since Smad4 also interacts with the first WW domain, there is a competitive binding interaction between Smad4 and Hyal-2 to WWOX.

High molecular weight HA increases the formation of endogenous Hyal-2/WWOX/Smad4 complex rapidly, followed by relocating to the nuclei in 20-40 min, in WWOX-expressing normal and cancer cells. No apparent cell death occurs. In WWOX-deficient cells, HA cannot effectively induce nuclear translocation of Smads, whereas Smad4 may spontaneously migrate to the nucleus. Stimulation of membrane Hyal-2 by specific antibody results in accumulation of WWOX and Smads in the nucleus. Furthermore, knockdown expression of Hyal-2 also increases nuclear accumulation of WWOX and Smads. These observations suggest that WWOX and Hyal-2 coordinate synergistically in relocating to the nucleus. Alternatively, WWOX and Hyal-2 may mutually sequester each other for nuclear relocation. WWOX is known to sequester Smad3 in the cytoplasm and inhibits its transcriptional function [48]. In a recent study, we determined that trafficking protein TRAPPC6A is a carrier for WWOX to undergo nuclear translocation [54–56]. Whether the TRAPPC6A/WWOX complex acts as a vehicle for Hyal-2 relocation to the nucleus remains to be established. In addition, whether WWOX interacts with AMOT (Angiomotin), a tight junction protein in the cell membrane/cytoskeletal area [27], during signaling remains to be determined.

To prove the HA-induced signaling event, we have carried out real time tri-molecular FRET analysis [42]. HA induces the signaling pathways for ectopic Smad4/WWOX/p53 and Smad4/Hyal-2/WWOX complexes. When the binding of Smad4, Hyal-2 and WWOX increases with time and the complex localizes in the nucleus, cells undergo bubbling cell death [51, 52]. Bubbling cell death is not apoptosis and is defined as “formation of a single nitric oxide-containing bubble from the nucleus per cell and release of this swelling bubble from the cell surface to extracellular space that causes cell death.” Bubbling cell death is caspase-independent, and exhibits no DNA fragmentation and flip-over of phosphatidylserine. In contrast, HA increases the binding affinity of Smad4/WWOX/p53 with time, and causes membrane blebbing but without cell death. We have designed the signaling in a reverse way. That is, the signaling starts from Smad4. In this manner, we know the complex has already formed in the cytoplasm and the signaling is working, which receives the signal from the upstream HA and membrane Hyal-2. We further showed the *in vivo* Hyal-2/WWOX complex accumulated in the apoptotic nuclei of neurons in the rat brains during traumatic brain injury. While native HA cannot induce cell death, HA may activate the overly expressed Smad4/Hyal-2/WWOX signaling complex and causes bubbling cell death.

Why does HA enhance cancer metastasis? HA constitutes approximately 20% of human body weight, and one third of HA undergoes degradation cycle on a daily basis [57, 58]. Degraded HA long and chain chains, from more than 20000 kDa down to oligomers, may promote cell growth and stimulate angiogenesis [1–8, 59, 60]. In most cases, malignant cancer cells are either devoid of tumor suppressors WWOX, Smad4 and p53 or possess mutations in these proteins, HA induced-signaling of Smad4/Hyal-2/WWOX and Smad4/WWOX/p53 for growth inhibition and death is blocked. This allows cancer cell growth advantage for metastasis [1–8]. Utilization of Smad proteins in the HA signaling supports the finding that HA either enhances or inhibits the TGF-β signaling [24, 61, 62]. We believe that both HA and TGF-β1 could simultaneously compete for utilization of Smads. We showed here that both HA and TGF-β1, together, may increase the nuclear localization of Hyal-2/WWOX/Smads complex.

WWOX is essential for neural development [34, 63]. Null mutation of *WWOX/Wwox* gene causes severe neural diseases and early death in humans and animals [22, 34]. HA protects neurons from traumatic brain injuries [64], and this could be related with HA-mediated disappearance of Hyal-2, which reduces the WWOX/Smad4 signaling. During embryonic development, murine WWOX is overexpressed in the cardiac cells and endothelia of blood vessels [63]. Also, high levels of WWOX are shown in the neural crest-derived structures such as cranial and spinal ganglia, skin pigment cells and mesenchyme in the head. The observations are in supporting with those studies in knockout animals and null mutations in humans, and suggest the key role of WWOX in neuronal development [22, 63–65].

## MATERIALS AND METHODS

### Cell lines

The following cell lines were maintained in our laboratory and used for indicated experiments: 1) murine TNF-sensitive L929 and TNF-resistant L929R fibroblasts, 2) human TβRII-deficient colorectal HCT116 cells, 3) human neuroblastoma SK-N-SH cells, 4) human prostate DU145 cells, 5) human breast ER^+^ WWOX^+^ MCF-7 cells, 6) human breast triple negative MDA-MB-231 and MDA-MB-435S cells, 7) human lung p53-deficient NCI-H1299 cells, and 8) monkey kidney COS7, 9) mouse breast cancer 4T1-Luc, 4T1-Luc-c1d, and 4T1-Luc-c5d cells, 10) human monocytic U937 and THP-1 cells, and 11) human T leukemia Jurkat and Molt-4 cells. These cells were from American Type Culture Collections (ATCC), Manassas, VA. 4T1-Luc-c1d, and 4T1-Luc-c5d cells were selected from cold shock challenge at 4°C for 1 and 5 days, respectively.

### Chemicals, antibodies and western blotting

Bovine testicular hyaluronidase PH-20, *Streptomyces hyalurolyticus* hyaluronidase, and human high-molecular-weight umbilical HA were from Sigma-Aldrich. Medical grade HA was from Lifecore Biomedical. (±)-Blebbistatin was from Calbiochem. We have generated antibodies against the *N-* and *C*-termini of WWOX [16, 37, 40], WWOX2 [19], Tyr33-phosphorylated WWOX [37], GST-WWOX [37], and Hyal-2 [21]. Commercial antibodies used were against: WWOX (N-19), JNK1, p53 (full length), Smad2, Smad3, Smad4, phospho-Smad2/3 (at Ser433/435), Tyr204-phosphorylated ERK (p-ERK) (the above antibodies from Santa Cruz Laboratories), phospho-Smad2 (Calbiochem), pS15-p53 (Calbiochem), pS46-p53 (R&D Systems), phospho-FAK (Upstate Biotechnology), and α-tubulin (Accurate Chemicals). Where indicated, Western blottings were carried out by our standard procedures [16, 17, 21, 23, 24, 23–25].

### Immunofluorescence microscopy and immunoelectron microscopy

DU145, COS7 and other indicated cells were cultured 48 hr on cover slides, fixed with formaldehyde (3.3%), permeabilized with 0.5% Triton-X100 (in the formaldehyde solution), and then stained with indicated primary antibodies and secondary fluorescent antibodies, followed by processing fluorescence microscopy [16, 19, 21, 23, 50]. Nuclei were stained with DAPI (4′,6-diamidino-2-phenylindole) (Calbiochem). Where indicated, immunoelectron microscopy was carried out as described [21, 40, 51]. Briefly, HCT116 cells were fixed for 1 hour using a freshly prepared 4% solution of paraformaldehyde in phosphate-buffered saline, immersed in 1% osmium tetroxide, dehydrated in a graded series of ethanol, and finally embedded in resin (EMS, SPUR's Kit). Ultrathin sections (70–80 nm) were prepared with an ultramicrotome (Reichert-Jung) and hybridized with an aliquot of IgG antibody against WWOX, p-WWOX, Smad4, or Hyal-2, followed by probing with a secondary anti-rabbit IgG 20 nm A-gold probe or anti-mouse (or goat) 10 nm gold particles. The sections were stained with saturated aqueous uranyl acetate and lead citrate at room temperature. Specimens were then observed under a transmission electron microscopy (JEOL JEM-1200EX, Japan) at 100 kV.

### cDNA expression constructs and transfection in cell lines and co-immunoprecipitation

The following expression constructs were made: 1) murine EGFP-WWOX [16], and 2) murine dominant-negative full length WWOX (dn-WWOX) and the *N*-terminal WW domain of WWOX (dn-WW), tagged with EGFP [16, 37]. The mutations were K28T and D29V. Murine Smad4 cDNA (from ATCC), encoding a truncated 43-kDa protein (GenBank accession AY493561) was tagged with in-frame with the *C*-terminal EGFP [21]. A plasmid construct was made in pSuppressorNeo vector for expressing siRNA to inhibit the expression of human and mouse WWOX protein [37].

Where indicated, COS7 or other cells were electroporated with the above constructs (200 volt, 50 msec; Square Wave BTX ECM830, Genetronics), cultured overnight, and then treated with hyaluronan and/or bovine hyaluronidase PH-20 (Sigma Chemicals) for indicated times, followed by preparing cytosolic and nuclear fractions [16, 21, 23, 37, 39, 41, 42]. The extent of protein expression was determined using specific antibodies. Co-immunoprecipitation was performed as described [16, 21, 23, 37, 39, 41, 42]. Briefly, SK-N-SH and other indicated cell lines were cultured overnight (in 150 mm Petri dishes) and then treated with HA (25 μg/ml) for 30 min, followed by processing immunoprecipitation using cytosolic and nuclear fractions. Where indicated, non-immune serum or IgG was used in negative controls.

### Cytoplasmic yeast two-hybrid analysis

Ras rescue-based yeast two-hybrid analysis (CytoTrap; Stratagene) was performed [16, 21, 37, 39, 42]. Briefly, activation of Ras signaling pathway in yeast occurs as a result of binding of a cytosolic Sos-tagged bait protein to a cell membrane-anchored target protein (tagged with a myristoylation signal). This activation allows mutant yeast cdc25H to grow in 37°C using a selective agarose plate containing galactose. Without binding, yeast cells fail to grow at 37°C. Target constructs made in a pMyr vector (with the myristoylation signal) were murine Smad4, murine Hyal-2, and human p53. Bait constructs made in a pSos vector (tagged with an *N*-terminal Sos protein) were murine WWOX, WWOXww (the first WW domain), WWOXww(Y33R), WWOXsdr (the entire SDR domain), WWOXsdr(mito) (the mitochondria-targeting region in the SDR domain), and WWOX(K28T/D29V) [16, 21, 37]. Additionally, constructs for a self-binding positive control were MafB (in both pMyr and pSos).

### Time lapse tri-molecular FRET microscopy

FRET microscopy was carried out [21, 23, 42]. DU145 cells were transiently overexpressed with each set of the following constructs: 1) ECFP-Smad4, EGFP-WWOX, DsRed-monomer-p53; 2) ECFP-Smad4, EGFP-Hyal-2(−sp), DsRed-monomer-WWOX; 3) ECFP-IκBα, EGFP-ERK, DsRed-monomer-WWOX [42]. Where indicated dominant negative WWOX(K28T/D29V) or p53(S46G) constructs was used to replace the wild type. FRET microscopy was performed using an inverted fluorescence microscope (Nikon Eclipse TE-2000U) and the data were analyzed as described.

### Image analysis and data presentation

We analyzed images by the NIH Image and Photoshop softwares. All experiments in this study were repeated 2-5 times.

## SUPPLEMENTARY MATERIALS FIGURES AND TABLES






















