# Supplementary material for: Hyaluronan activates Hyal-2/WWOX/Smad4 signaling and causes bubbling cell death when the signaling complex is overexpressed
Source: Oncotarget. 2016 Nov 10;8(12):19137–55. doi: 10.18632/oncotarget.13268 (PMC5386674; doi:10.18632/oncotarget.13268)
Supplement: Supplementary file 1 [file oncotarget-08-19137-s001.pdf]

Hyaluronan activates Hyal-2/WWOX/Smad4 signaling and causes bubbling cell death when the signaling complex is overexpressed

SUPPLEMENTARY FIGURES AND VIDEOS

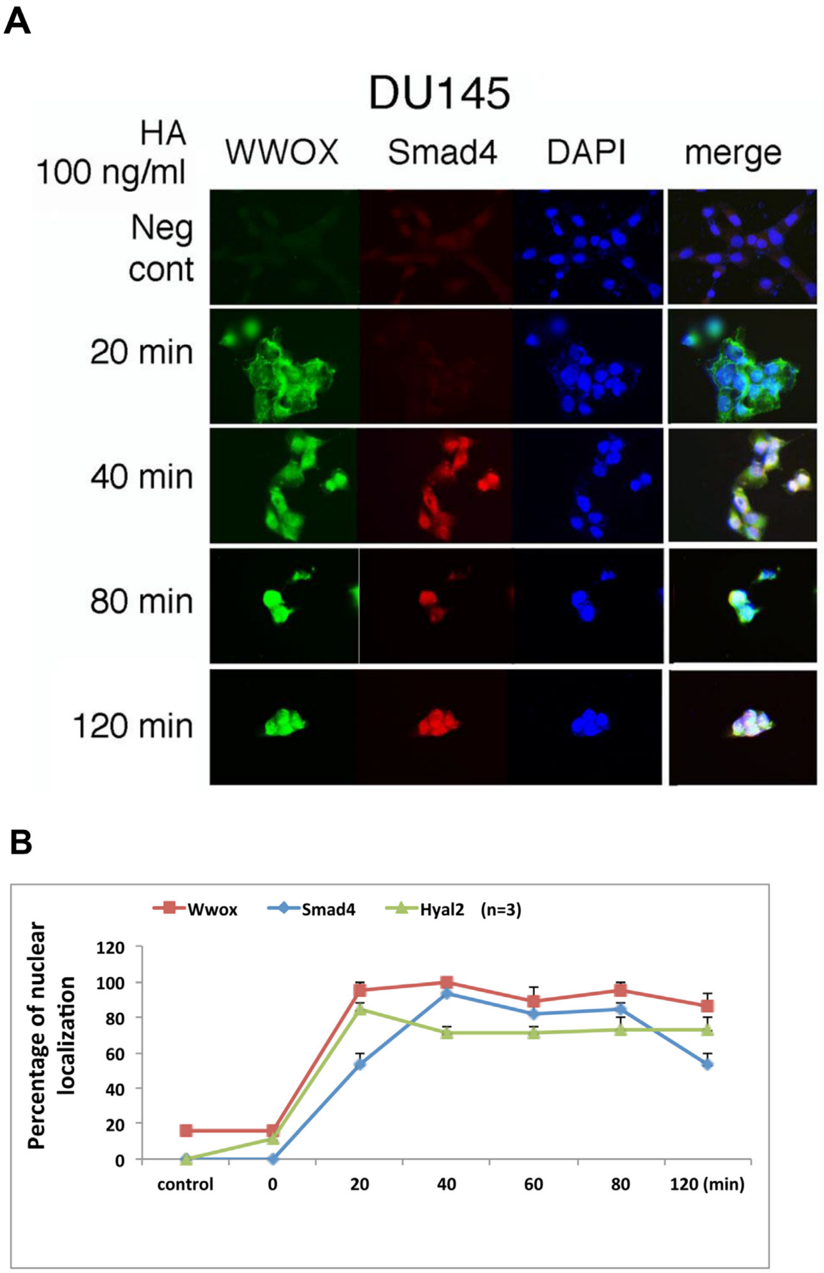

Supplementary Figure S1: HA induces relocation of WWOX, Hyal-2 and Smad4 to the nuclei in prostate cancer DU145 cells. A. DU145 cells were treated with a low level of high molecular weight HA (100 ng/ml) for indicated times. Nuclear accumulation of WWOX and Smad4 is shown. **B.** Shown is a quantified data for nuclear localization of WWOX, Hyal-2 and Smad4 (mean standard ± deviation; n=3; 20 cells per count).

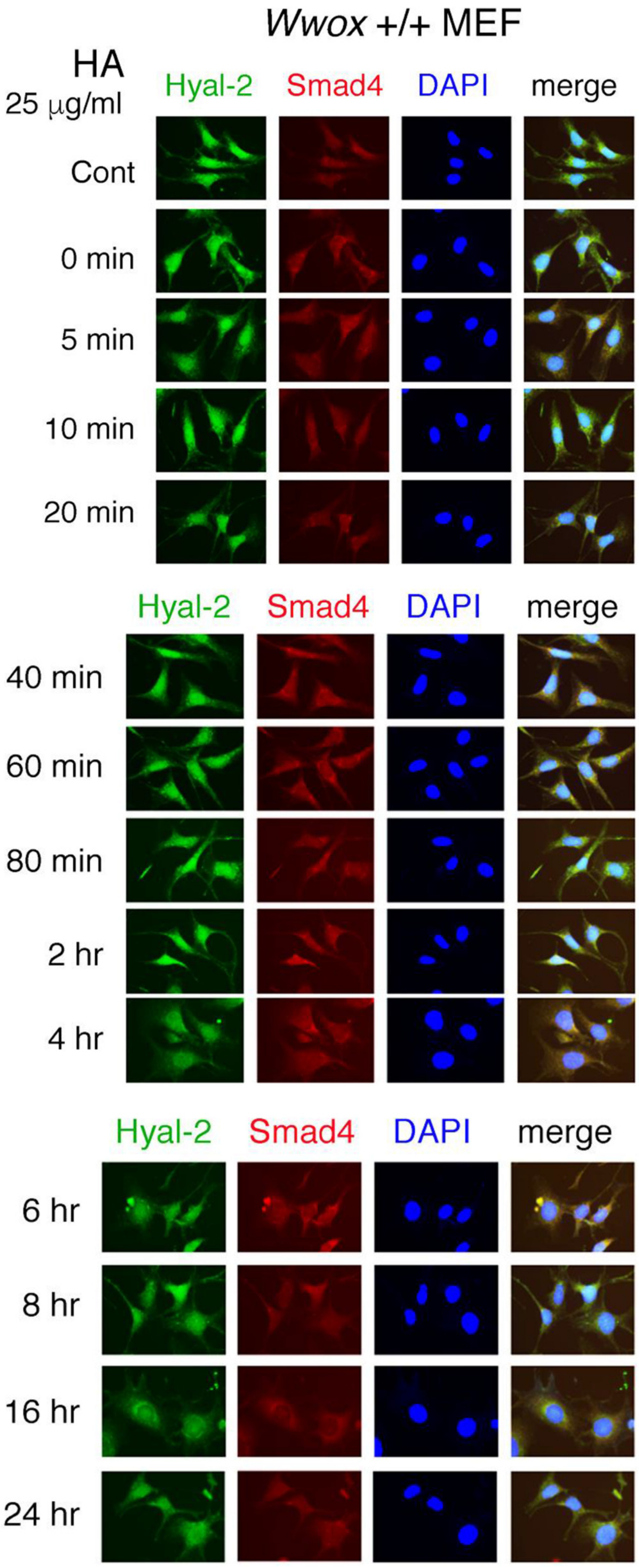

Supplementary Figure S2: HA rapidly induces relocation of Hyal-2 and Smad4 to the nuclei in *Wwox*$^{+/+}$ MEF cells. Cells were treated with high molecular weight HA (25 μg/ml) for indicated times. Note the rapid nuclear localization of Hyal-2 and Smad4 in 5 min (also see Figures 2D and 4C).

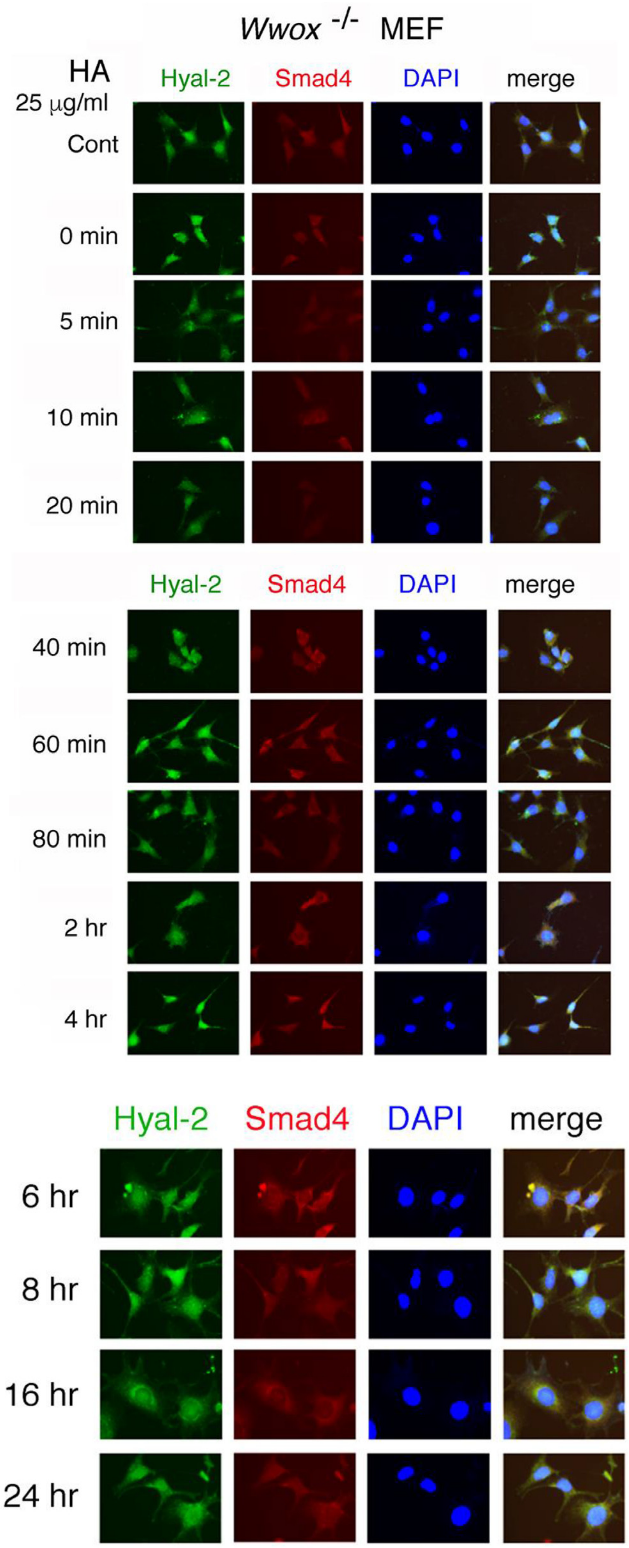

Supplementary Figure S3: Knockout *Wwox*$^{-/-}$ MEF cells are refractory to HA-induced relocation of Hyal-2 and Smad4 to the nuclei. Cells were treated with high molecular weight HA (25 μg/ml) for indicated times. Endogenous Smad4 did not appear to relocate into nucleus (also see Figure 2D). Around 30% of the cells have nuclear localization of Hyal-2, and HA reduced the localization with time (also see Figure 4C).

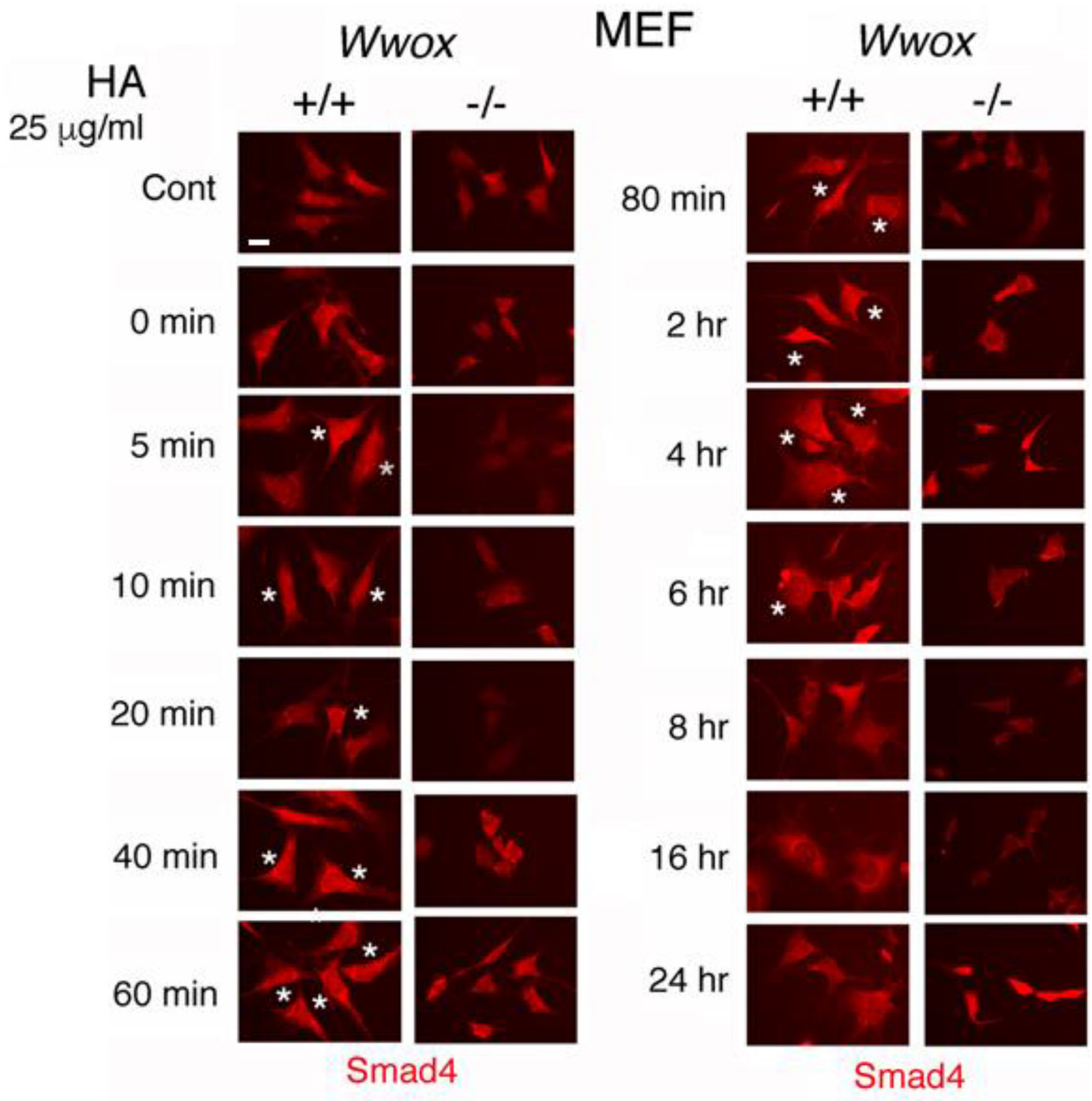

Supplementary Figure S4: HA induction of Smad4 translocation to the nucleus in *Wwox* MEF cells. When wild type *Wwox*$^{+/+}$ MEF cells were exposed to HA (25 μg/ml) for indicated times, cytosolic Smad4 relocated into the nucleus in 5 min. However, it took about 4 hr for Smad4 to relocate into nucleus in knockout *Wwox*$^{-/-}$ MEF cells. Scale bar, 10 μm. See the quantification data in the Figure 2D.

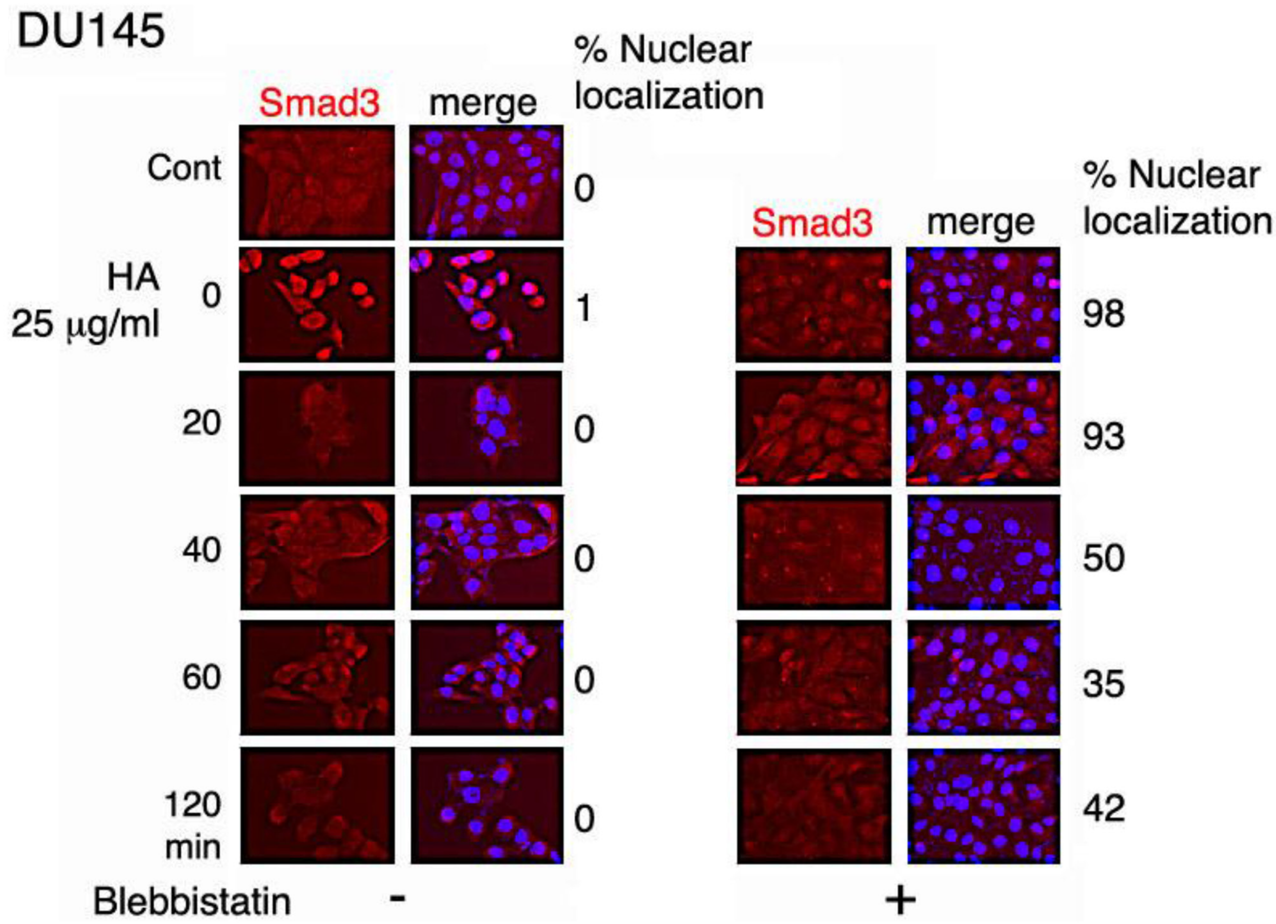

Supplementary Figure S5: Blebbistatin induces Smad3 translocation to the nuclei in DU145 cells. DU145 cells were pre-treated with or without blebbistatin (20 μM) for 30 min, followed by exposure to HA for indicated times. Note that HA had no effect in inducing nuclear translocation of Smad3. In stark contrast, blebbistatin rapidly caused Smad3 to migrate to the nucleus during pre-treatment for 30 min.

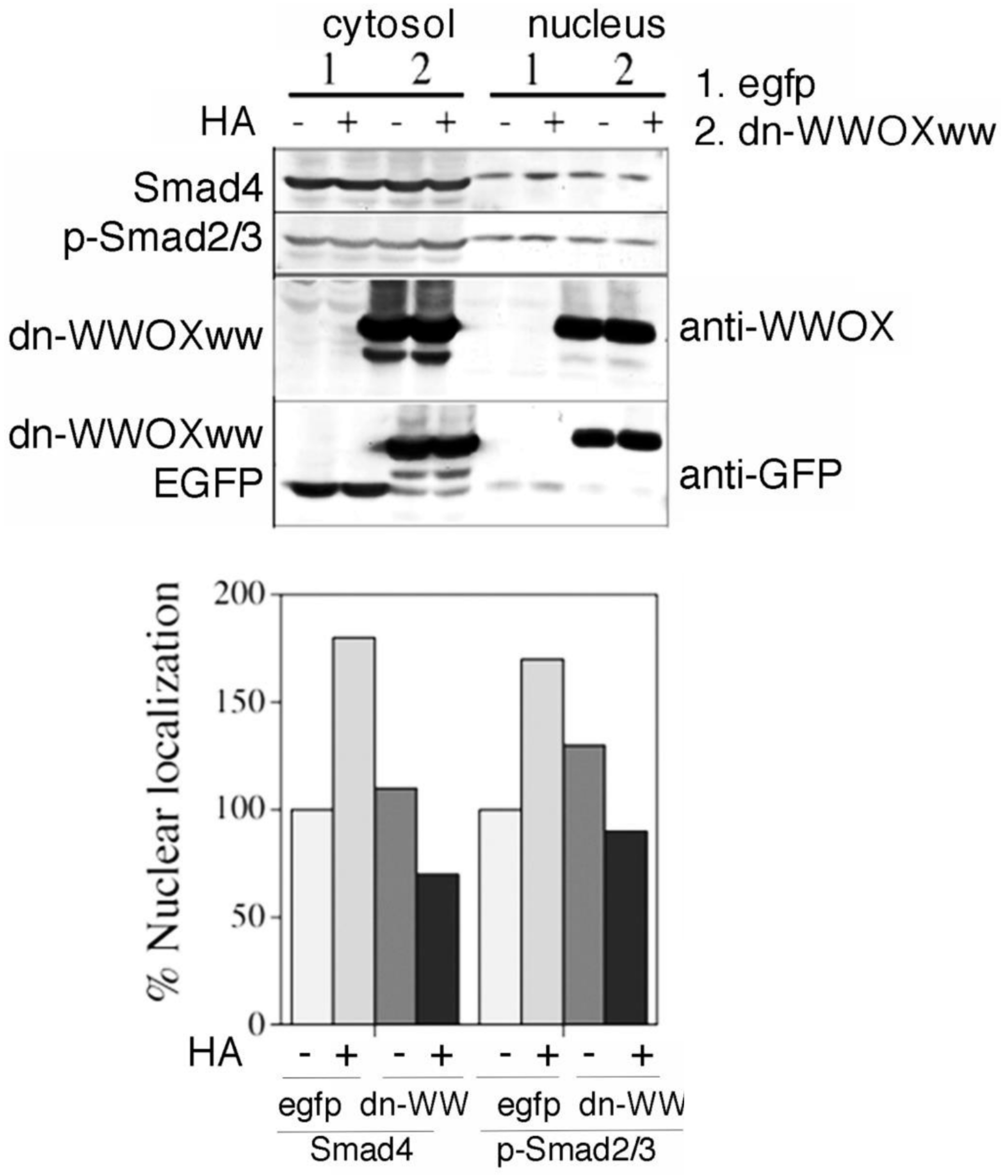

Supplementary Figure S6: Dominant negative WWOX (dn-WWOX) blocks HA-induced phosphorylation of Smad2/3. COS7 cells were transfected with dn-WWOXww (at the first WW domain) or EGFP alone, cultured 48 hr, and then treated with HA for 30 min. dn-WWOXww blocked HA-induced phosphorylation of Smad2/3 at the nuclear level, and inhibited nuclear translocation of Smad4 (top panel). The expressed ectopic proteins are shown in the last 2 rows from bottom. The extent of protein translocation was quantified (average from two experiments; bottom panel).

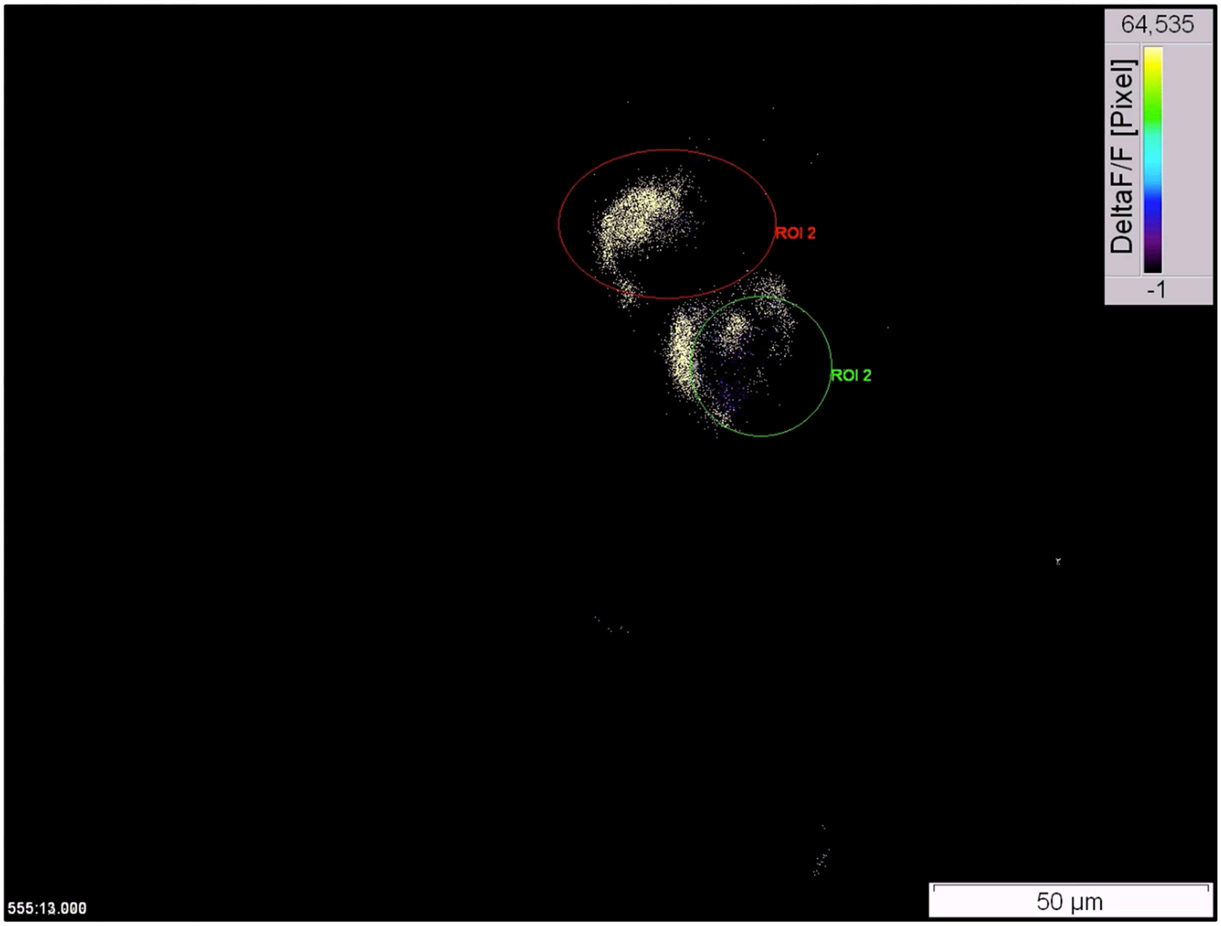

Supplementary Video 1: Smad4/WWOX/p53 signaling. DU145 cells were transiently transfected with ECFP-Smad4, EGFP-WWOX and DsRed-monomer-p53 expression constructs. After culturing overnight, cells were treated with HA (25 µg/ml). Time-lapse FRET microscopy for interactions of 3 proteins was carried out [42]. Each picture was taken per 20 min. Data is shown as FRETc (FRET concentration).

See Supplementary video 1

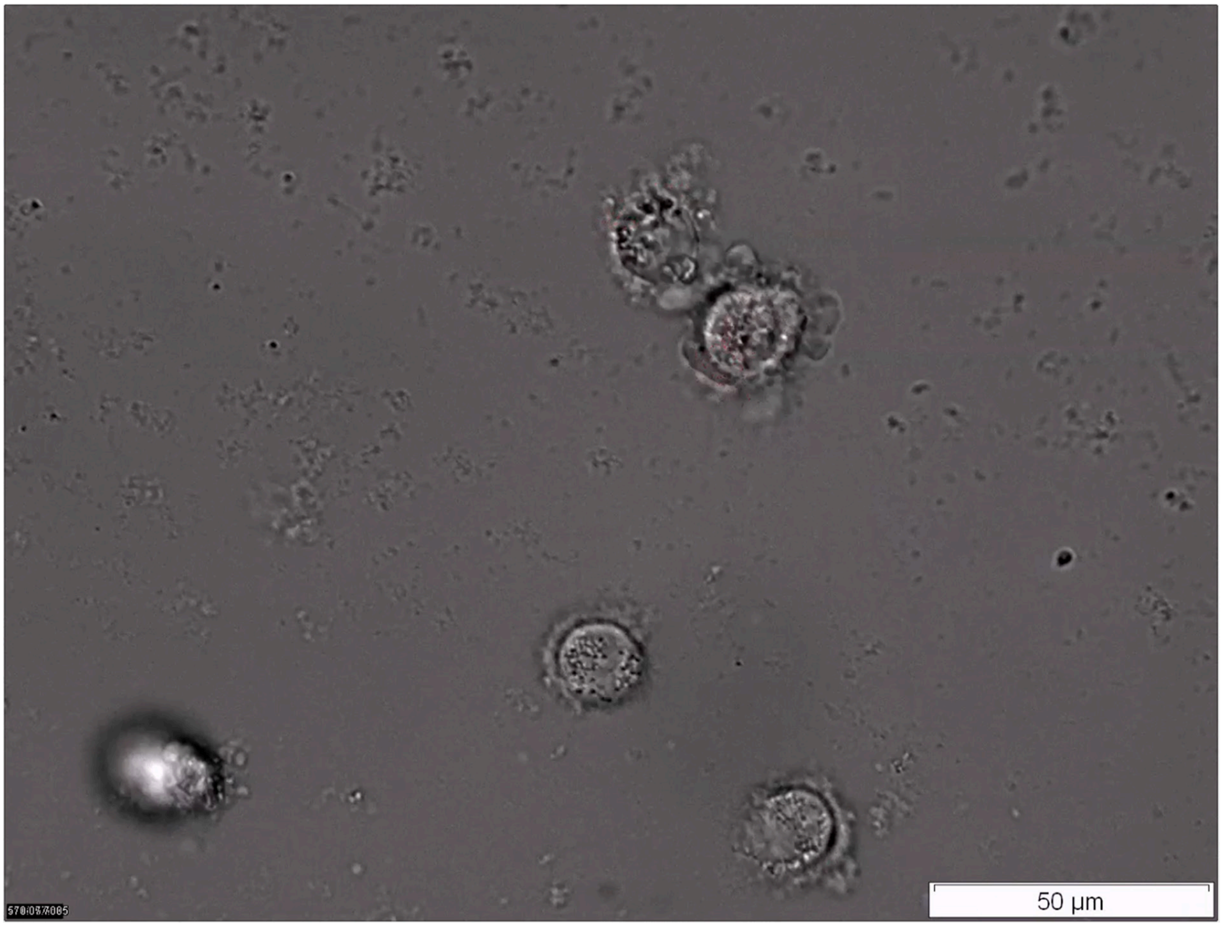

Supplementary Video 2: Smad4/WWOX/p53 signaling – merged images of FRETc and bright field. From the above experiment (Video 1), images of FRETc and bright field were merged. False positive signals were due to floating dead cells. (This may be seen in other videos)

See Supplementary video 2

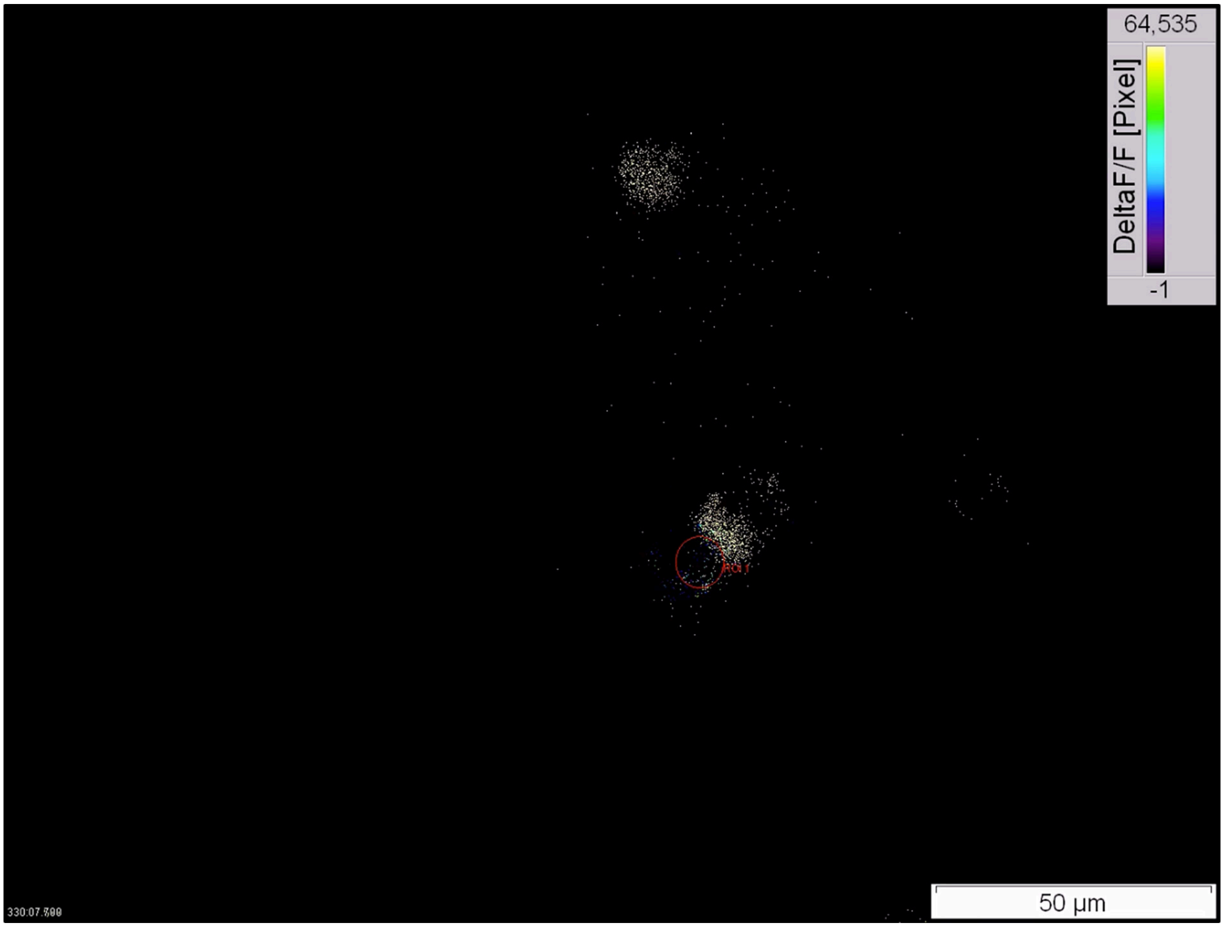

Supplementary Video 3: Smad4/dn-WWOX/p53 signaling. DU145 cells were transiently transfected with ECFP-Smad4, dn-EGFP-WWOX and DsRed-monomer-p53 expression constructs. After culturing overnight, cells were treated with HA (25 μg/ml). Time-lapse FRET microscopy for interactions of 3 proteins was carried out [42]. Each picture was taken per 20 min. Data is shown as FRETc (FRET concentration). No signaling occurred.

See Supplementary video 3

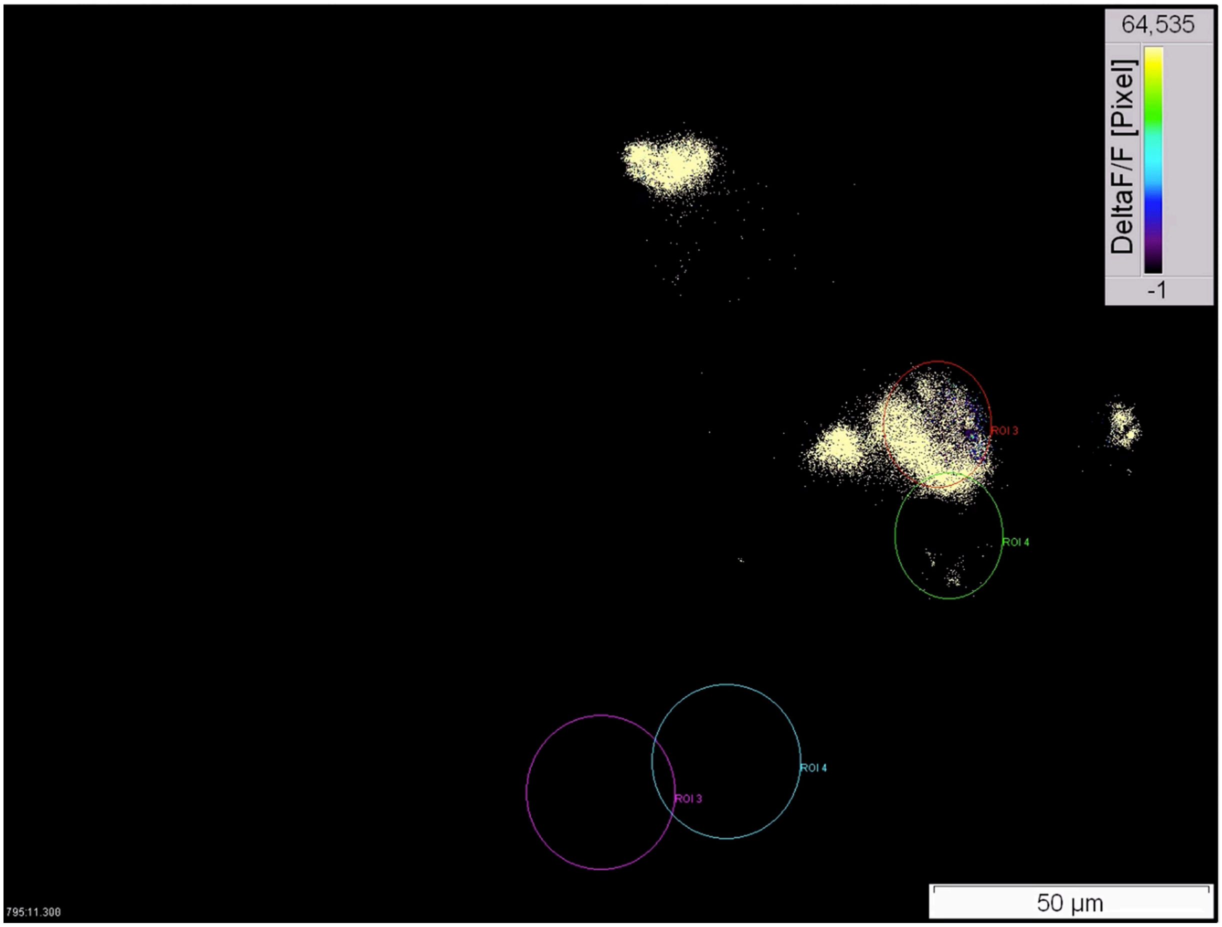

Supplementary Video 4: Smad4/WWOX/dn-p53 signaling. DU145 cells were transiently transfected with ECFP-Smad4, EGFP-WWOX and DsRed-monomer-dn-p53 [or p53(S46G)] expression constructs. After culturing overnight, cells were treated with HA (25 µg/ml). Time-lapse FRET microscopy for interactions of 3 proteins was carried out [42]. Each picture was taken per 20 min. Data is shown as FRETc (FRET concentration). No signaling occurred.

See Supplementary video 4

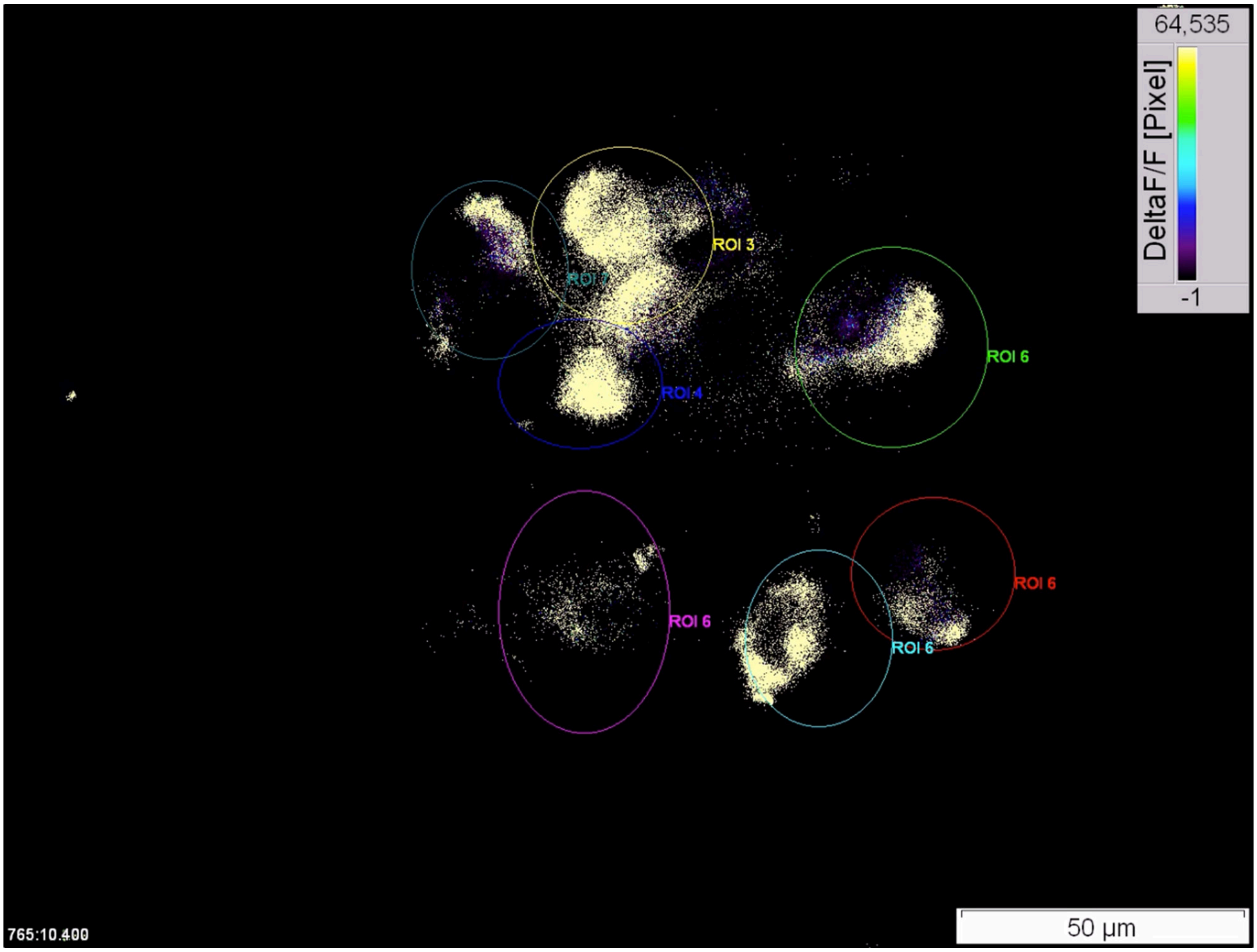

Supplementary Video 5: Smad4/Hyal-2(-sp)/WWOX signaling. DU145 cells were transiently transfected with ECFP-Smad4, EGFP-Hyal-2(-sp) and DsRed-monomer-WWOX expression constructs. After culturing overnight, cells were treated with HA (25 µg/ml). Time-lapse FRET microscopy for interactions of 3 proteins was carried out [42]. Each picture was taken per 20 min. Data is shown as FRETc (FRET concentration). EGFP-Hyal-2(-sp) does not have a GPI sequence and is for cytosolic protein expression.

See Supplementary video 5

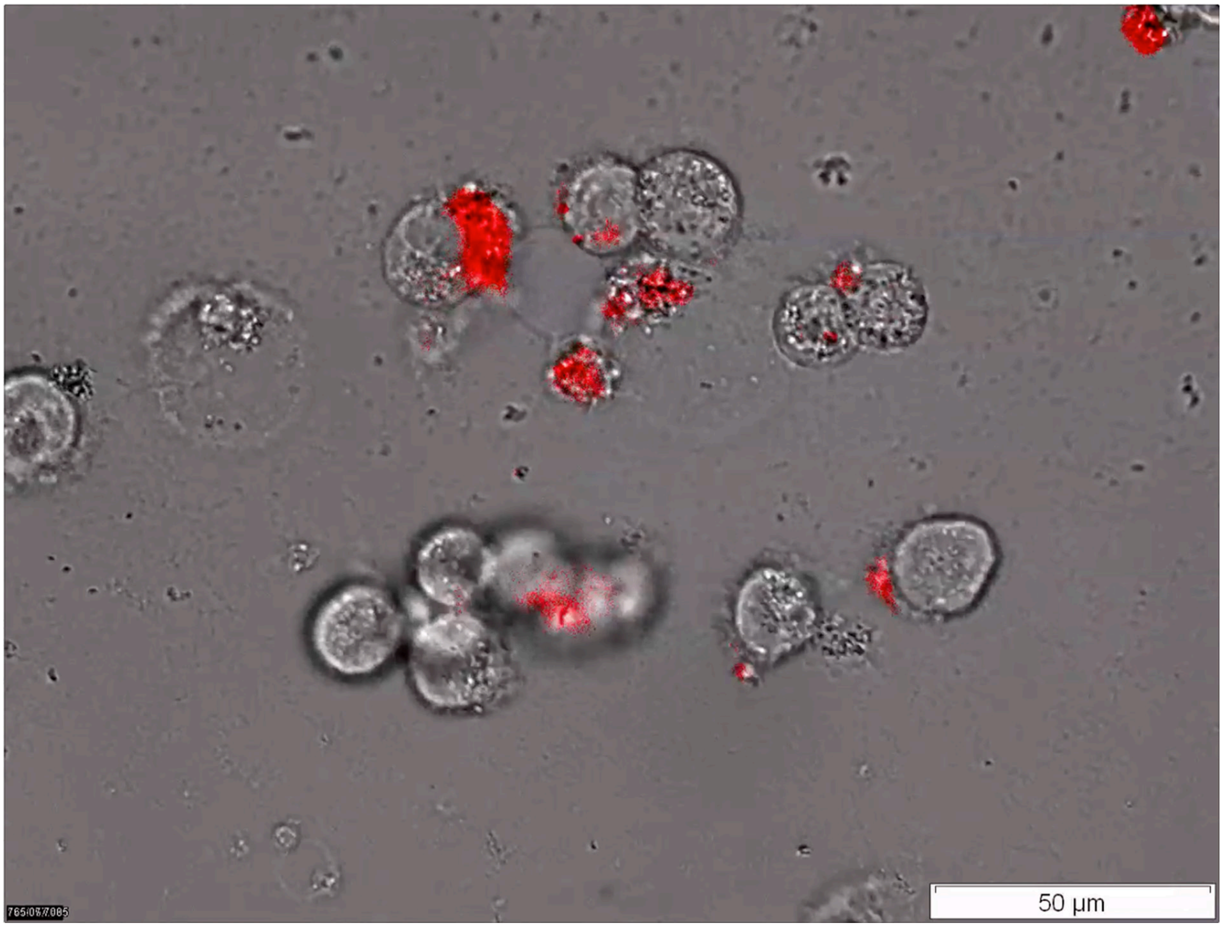

Supplementary Video 6: Smad4/Hyal-2(-sp)/WWOX signaling – merged images of FRETc and bright field. From the above experiment (Video 5), images of FRETc and bright field were merged.

See Supplementary video 6

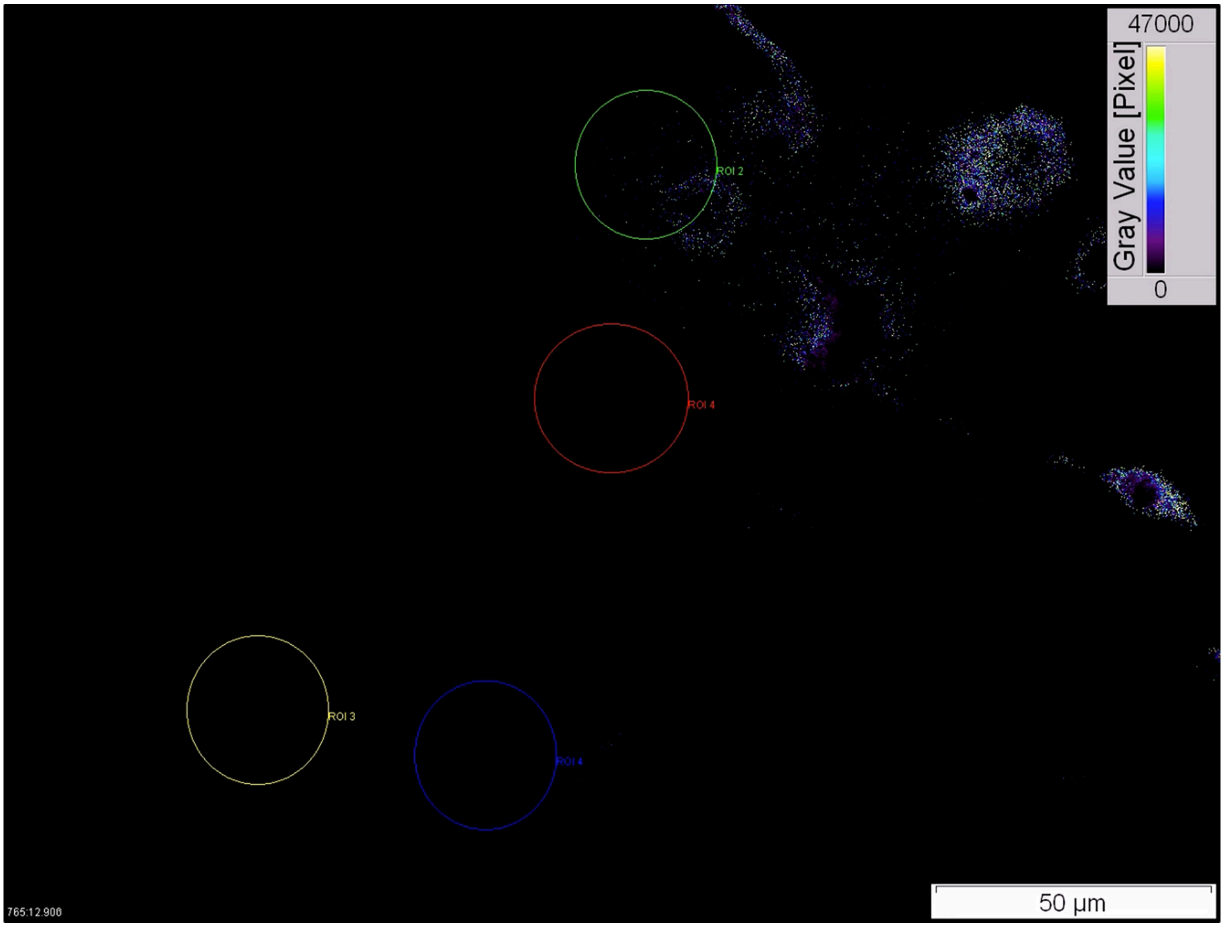

Supplementary Video 7: Smad4/Hyal-2(as)/WWOX signaling. DU145 cells were transiently transfected with ECFP-Smad4, EGFP-Hyal-2(as) and DsRed-monomer-WWOX expression constructs. EGFP-Hyal-2(as) is an antisense construct. After culturing overnight, cells were treated with HA (25 µg/ml). Time-lapse FRET microscopy for interactions of 3 proteins was carried out [42]. Each picture was taken per 20 min. Data is shown as FRETc (FRET concentration). No signaling occurred.

See Supplementary video 7

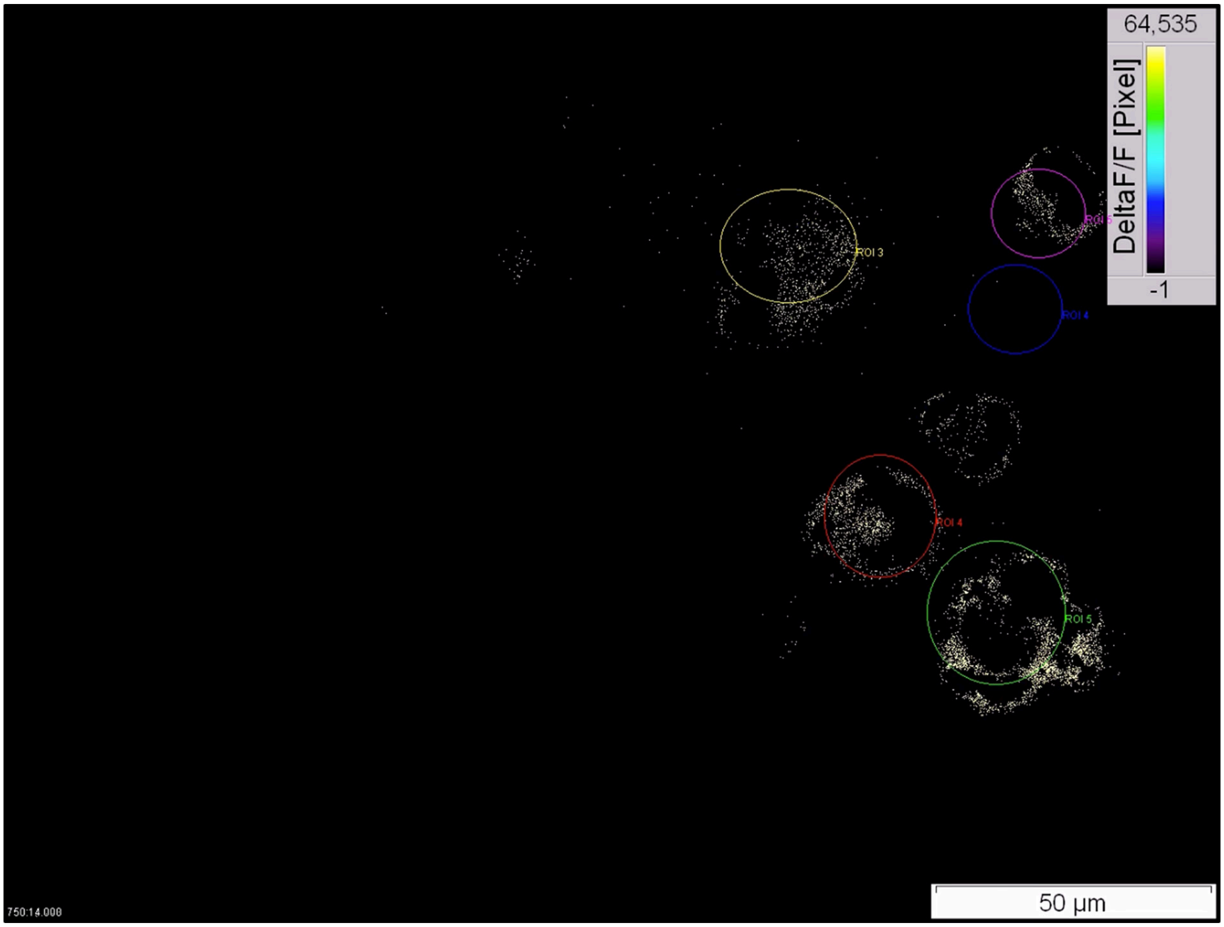

Supplementary Video 8: Smad4/Hyal-2(-sp)/dn-WWOX signaling. DU145 cells were transiently transfected with ECFP-Smad4, EGFP-Hyal-2(-sp) and DsRed-monomer-dn-WWOX expression constructs. After culturing overnight, cells were treated with HA (25 µg/ml). Time-lapse FRET microscopy for interactions of 3 proteins was carried out [42]. Each picture was taken per 20 min. Data is shown as FRETc (FRET concentration). No signaling occurred.

See Supplementary video 8

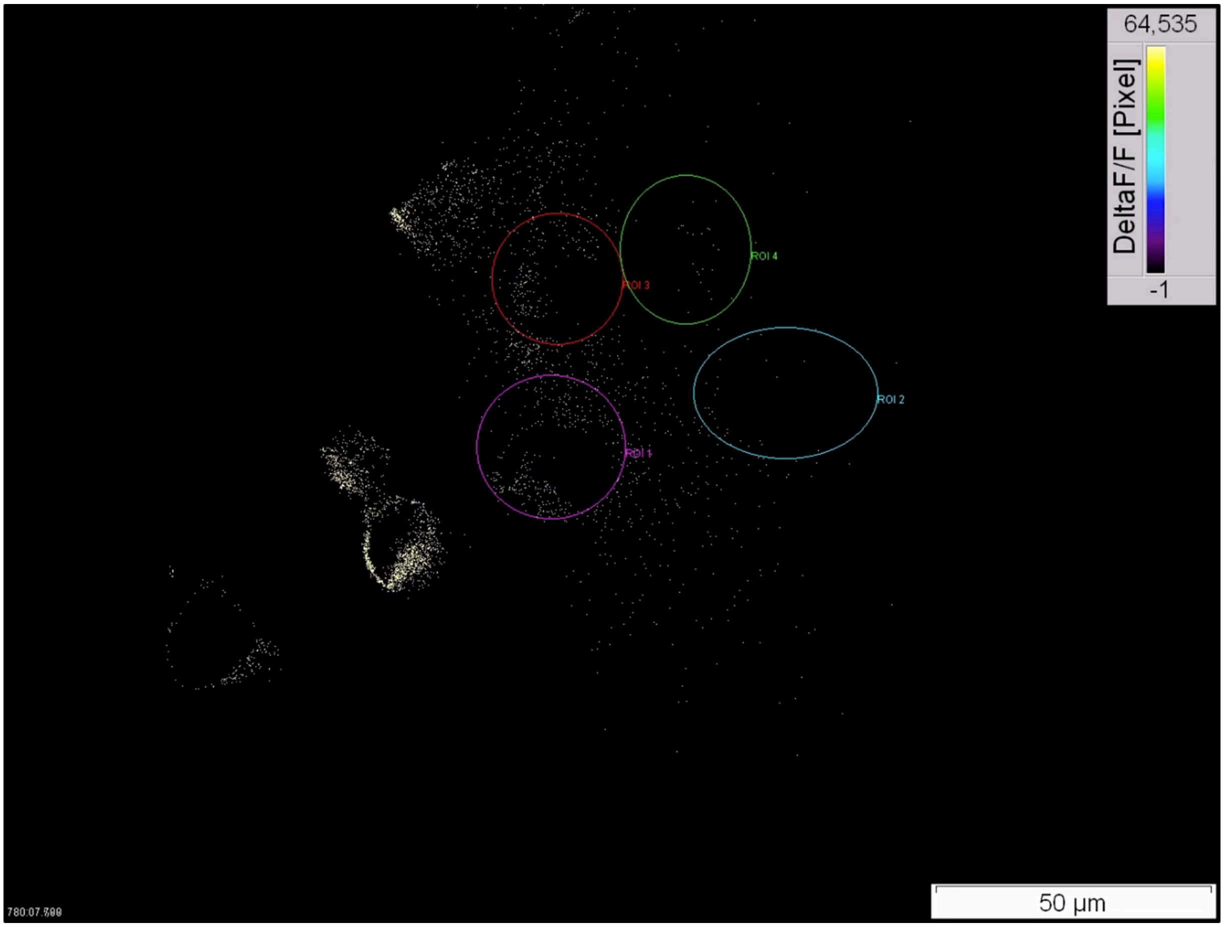

Supplementary Video 9: ECFP/EGFP/DsRed monomer signaling. DU145 cells were transiently transfected with ECFP, EGFP and DsRed-monomer expression constructs. After culturing overnight, cells were treated with HA (25 µg/ml). Time-lapse FRET microscopy for interactions of 3 proteins was carried out [42]. Each picture was taken per 20 min. Data is shown as FRETc (FRET concentration). No signaling occurred.

See Supplementary video 9

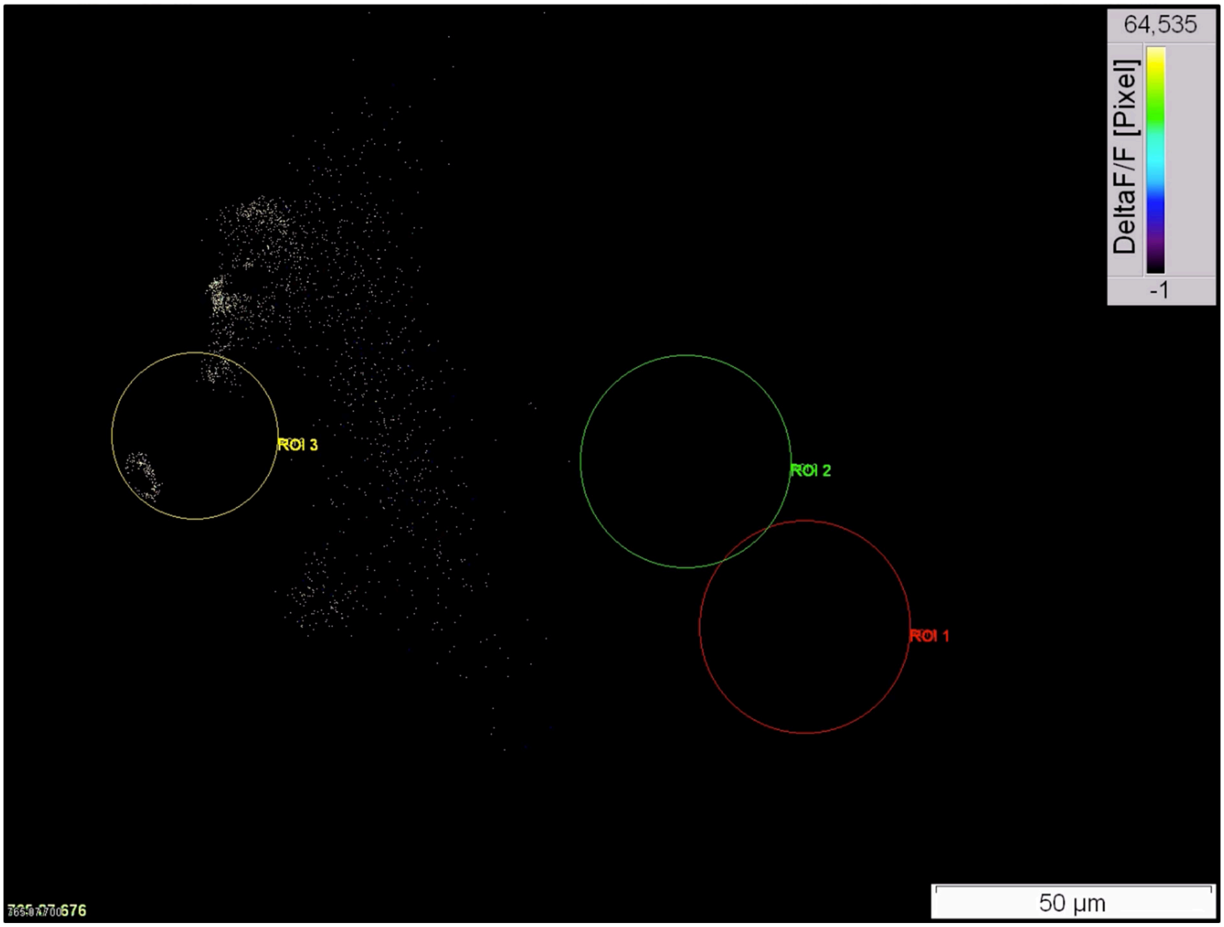

Supplementary Video 10: IκBα/ERK/WWOX signaling. DU145 cells were transiently transfected with ECFP-IκBα, EGFP-ERK and DsRed-monomer-WWOX expression constructs. After culturing overnight, cells were treated with HA (25 µg/ml). Time-lapse FRET microscopy for interactions of 3 proteins was carried out [42]. Each picture was taken per 20 min. Data is shown as FRETc (FRET concentration). No signaling occurred.

See Supplementary video 10
